# Single-cell and bulk RNA-seq unveils the immune infiltration landscape associated with cuproptosis in cerebral cavernous malformations

**DOI:** 10.1186/s40364-024-00603-y

**Published:** 2024-06-05

**Authors:** Chengwei Chen, Yuting Bao, Sihan Ju, Conglin Jiang, Xiang Zou, Xin Zhang, Liang Chen

**Affiliations:** 1 Neurosurgical department of Huashan hospital and MOE Frontiers Center for Brain Science, Fudan University, Shanghai, 200040 China; 2 Tianqiao and Chrissy Chen Institute Clinical Translational Research Center, Shanghai, 200040 China; 3 Research Unit of New Technologies of Micro-Endoscopy Combination in Skull Base Surgery (2018RU008), Chinese Academy of Medical Sciences, Beijing, China; 4 National Center for Neurological Disorders, Shanghai, 200040 China; 5 Shanghai Key Laboratory of Brain Function Restoration and Neural Regeneration, Shanghai, 200040 China; 6 Neurosurgical Institute of Fudan University, Shanghai, 200040 China; 7 Shanghai Clinical Medical Center of Neurosurgery, Shanghai, 200040 China; 8 Neurosurgery Center, Department of Cerebrovascular Surgery, The National Key Clinical Specialty, The Engineering Technology Research Center of Education Ministry of China on Diagnosis and Treatment of Cerebrovascular Disease, Guangdong Provincial Key Laboratory on Brain Function Repair and Regeneration, The Neurosurgery Institute of Guangdong Province, Zhujiang Hospital, Southern Medical University, Guangzhou, 510282 China

**Keywords:** Cerebral cavernous malformations, Cuproptosis, Immune infiltration, Cuproptosis-related genes, Cellchat

## Abstract

**Background:**

Cerebral cavernous malformations (CCMs) are vascular abnormalities associated with deregulated angiogenesis. Their pathogenesis and optimal treatment remain unclear. This study aims to investigate the molecular signatures of cuproptosis, a newly identified type of cell death, associated with CCMs development.

**Methods:**

Bulk RNA sequencing (RNA-seq) from 15 CCM and 6 control samples were performed with consensus clustering and clustered to two subtypes based on expression levels of cuproptosis-related genes (CRGs). Differentially expressed genes and immune infiltration between subtypes were then identified. Machine learning algorithms including the least absolute shrinkage and selection operator and random forest were employed to screen for hub genes for CCMs associated with cuproptosis. Furthermore, Pathway enrichment and correlation analysis were used to explore the functions of hub genes and their association with immune phenotypes in CCMs. An external dataset was then employed for validation. Finally, employing the Cellchat algorithm on a single-cell RNA-seq dataset, we explored potential mechanisms underlying the participation of these hub genes in cell-cell communication in CCMs.

**Results:**

Our study revealed two distinct CCM subtypes with differential pattern of CRG expression and immune infiltration. Three hub genes (BTBD10, PFDN4, and CEMIP) were identified and validated, which may significantly associate with CCM pathogenesis. These genes were found to be significantly upregulated in CCM endothelial cells (ECs) and were validated through immunofluorescence and western blot analysis. Single-cell RNA-seq analysis revealed the cellular co-expression patterns of these hub genes, particularly highlighting the high expression of BTBD10 and PFDN4 in ECs. Additionally, a significant co-localization was also observed between BTBD10 and the pivotal cuproptosis gene FDX1 in Mki67+ tip cells, indicating the crucial role of cuproptosis for angiogenesis in CCMs. The study also explored the cell-cell communication between subcluster of ECs expressing these hub genes and immune cells, particularly M2 macrophages, suggesting a role for these interactions in CCM pathogenesis.

**Conclusion:**

This study identifies molecular signatures linking cuproptosis to CCMs pathogenesis. Three hub genes—PFDN4, CEMIP, and BTBD10—may influence disease progression by modulating immunity. Further research is needed to understand their precise disease mechanisms and evaluate their potential as biomarkers or therapeutic targets for CCMs.

**Supplementary Information:**

The online version contains supplementary material available at 10.1186/s40364-024-00603-y.

## Background

Cerebral cavernous malformations (CCMs) are prevalent neurovascular malformations that occur in either sporadic or familial forms in young adults [1–3]. The sporadic form is frequently linked with a developmental venous anomaly and has a single focus, while familial cases are characterized by the presence of multiple lesions [4]. These malformations have been extensively researched, but their origins and prevalence in different age groups are still under consideration. CCMs are characterized by abnormally dilated blood vessels in the venous-capillary vascular bed without intervening brain parenchyma [1, 5]. The blood contained in such vessels moves slowly and tends to clot [6, 7]. The lesions are associated with a lifelong risk of stroke, seizures, and focal neurological deficits for which no effective pharmacological treatment has been confirmed [8, 9]. To date, three genes have been identified to be responsible for development of CCMs, namely CCM1 (KRIT1), CCM2 (MGC4607), and CCM3 (PDCD10) [10–13]. The reported mutations of these three genes reported so far result in premature termination codons or large deletions, indicating loss-of-function mutations involved in the formation of enlarged thin-walled vessels [13–16]. It has been discovered that genes and genetic risk factors are involved in vasculogenesis, angiogenesis, and vascular remodeling [17–19]. However, the precise mechanisms lead to the pathogenesis of CCMs are still unclear, the therapeutic approaches have yet to be determined.

Cuproptosis, also identified as copper-induced death, a novel form of cell death associated with mitochondrial metabolism that distinctly diverges from other known forms of death including apoptosis, ferroptosis and necroptosis [20]. Recently, the reliance of cuproptosis on mitochondrial respiration stimulated by either deficient or excess intracellular copper has been established. Copper insufficiency impairs the function of copper-binding enzymes [21]. The overabundance of copper inside cells can be transported towards the mitochondria, where it directly binds with lipid-acylated components within the tricarboxylic acid (TCA) cycle, initiating toxic protein stress and ultimately resulting in cell death [22]. Based on a whole-genome CRISPR-Cas9 screening, several cuproptosis-related genes (CRGs) have been identified [22]. Among these, seven genes, namely ferredoxin 1 (FDX1), lipoic acid synthase (LIAS), lipoyltransferase 1 (LIPT1), dihydrolipoamide dehydrogenase (DLD), dihydrolipoamide S-acetyltransferase (DLAT), pyruvate dehydrogenase E1 component subunit alpha 1 (PDHA1) and pyruvate dehydrogenase beta subunit (PDHB) have exhibited positive regulatory effects on cuproptosis, while three genes including metal regulatory transcription factor 1 (MTF1), glutaminase (GLS), and cyclin-dependent kinase inhibitor 2A (CDKN2A), have demonstrated negative regulatory effects. Moreover, maintaining the intracellular copper concentration is reliant on copper exporters and importers [22, 23]. Genetic mutations that lead to copper accumulation are associated with severe and potentially life-threatening pathological conditions, including Menke’s disease [24], Wilson’s disease [25], neurodegenerative diseases [26, 27], cancer [28], and cardiovascular disease [29]. Copper is demonstrated to be involved in the development and progression of cancer by triggering cell proliferation, angiogenesis, and metastasis [20]. CCMs are collections of abnormal slow-flow capillaries predominantly found in the central nervous system, characterized by dilated, thin-walled vessels predisposed to recurrent hemorrhages within the malformed vascular cluster [30]. Although there is currently no direct evidence indicating the involvement of copper metabolism in the progression of CCMs, copper has been found to demonstrate pro-angiogenic properties by regulating various factors involved in angiogenesis [31–33]. Furthermore, copper levels play a role in cardiovascular diseases, with higher levels in blood associated with increased risk [34]. Disturbance of copper homeostasis can also result in mitochondrial dysfunction, leading to vascular endothelial cells (ECs) injury, affecting vascular development, and potentially contributing to hemorrhage [35]. Evidence also indicates that copper cytotoxicity may potentially contribute to vascular endothelial injury in vascular diseases [36]. Abnormal lipoylation protein oligomerization, known to be a key process in cuproptosis, has been identified as playing a role in endothelial damage [22, 37]. Disruption of the lipoylation pathway has been demonstrated to lead to mitochondrial dysfunction in ECs, impairing vascular growth and development in mouse models[37]. Studies have also shown that α-lipoic acid-plus exerts endovascular protective effects by reducing mitochondrial damage and helping to maintain lysosomal integrity, inhibiting the apoptosis pathway post intimal injury [38, 39]. Considering the roles of copper metabolism and aberrant protein lipoylation in the vascular endothelium, along with the characteristics of abnormal vascular development and a tendency for recurrent hemorrhages in CCMs, we are speculating on whether cuproptosis are involved in the pathogenesis and development of CCMs. Therefore, investigate the relationship between CRGs and CCMs, which may provide insight into potential therapeutic targets.

In this study, we use a comprehensive approach to investigate the potential involvement cuproptosis in the progression of CCMs. We explore the contribution of CRGs in different subtypes of CCMs, screen for hub genes related to cuproptosis, and validate these genes in an external dataset and CCM samples from our cohort. Furthermore, our study has explored the intricate interplay between these hub genes and the immune microenvironment within CCMs on single-cell level, providing novel insights into the role of immune modulation in the disease process. Our result may provide valuable insight into the diverse and complex mechanisms underlying CCMs development and progression, further paving the way for potential therapeutic strategies targeting cuproptosis-associated factors.

## Methods

### Study design

**Fig. 1:**
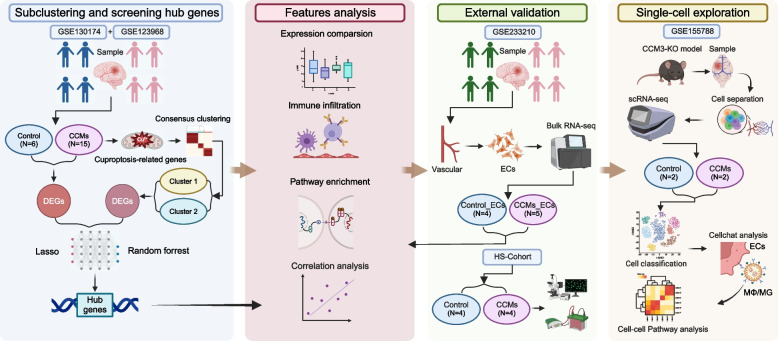
Flow chart of the study. Created with Biorender.com

The study design is presented in Fig. 1.

### Bulk RNA Sequencing (RNA-seq ) data collection

Three datasets were retrieved from the Gene Expression Omnibus (GEO) database, including three bulk RNA-seq datasets (GSE130174, GSE123968, and GSE233210). Dataset GSE130174, which comprised 10 CCMs samples and three control samples, was annotated using platform GPL20301. Similarly, dataset GSE123968, also annotated using platform GPL20301, comprised five CCMs samples and three control samples. For subtyping of CCMs based on expression of CRGs and identification of hub genes, we integrated GSE130174 and GSE123968 using the Combat function of the "sva" R package. The GSE233210 dataset, annotated using platform GPL24676, which included five sorted EC samples from CCMs patients and four normal control samples, was used as an external validation dataset.

### Consensus clustering analysis

The human bulk RNA-seq datasets GSE130174 and GSE123968 were combined for primary analysis. Consensus clustering analysis was performed with “ConsensusClusterPlus” package. Based on the expression levels of CRGs, CCMs were stratified into clusters through consensus clustering involving 50 iterations, each consisting 80% of the samples. The optimal number of clusters was identified using cumulative distribution function curves of consistency scores and the consistency matrix heat map features of the consistency matrix.

### Differential expression genes (DEGs) analysis

We employed the “Limma” package, a specialized R package for analyzing differential expression of genetic profiles, to identify the genes which display significant differential expression across distinct comparison groups to investigate the variations in the molecular mechanisms of CCMs data. DEGs were identified between the control and CCMs samples, as well as between subtypes of CCMs clustered based on the expression of CRGs. Only those genes with a *P* < 0.05 and |log2FC| > 1 were selected. The DEGs were then used to formulated volcano plots with the “ggplot2” package to visual the distribution. DEGs were also utilized Metascape (https://metascape.org/) for Gene Ontology (GO) enrichment analysis to determine the major biological terms.

### Machine learning to identify CRGs associated hub genes in CCMs subtypes

This study employed the least absolute shrinkage and selection operator (LASSO) regression and random forest algorithms to identify hub genes associated with CRGs in different molecular subtypes of CCMs. The gene set for machine learning were selected from the intersection of the DEGs from different subtypes of CCMs and the DEGs from the disease and normal control groups (Fig. 1). The LASSO algorithm was executed using the "glmnet" package, while the random forest algorithm utilized integrated learning with decision trees as base learners. Training sets were selected from the sample set with replacement, and decision trees were generated from the resulting samples. Features are randomly and non-repetitively selected at each node, and subsequently used to divide the sample set to identify the optimal feature for division and prediction. In this study, feature importance was evaluated by the random forest algorithm, which involved the construction of 1000 classification trees, each of which was churned 50 times to assess the importance of the features based on %IncMSE. The hub genes were obtained by taking the intersection of the results from Lasso regression and random forest.

### Immune infiltration analysis

The single‑sample gene set enrichment analysis (ssGSEA) technique is widely implemented for assessing the different immune cell types present in the microenvironment. It is founded on the support vector regression principle and back-convolution enquiry of the immune cell subtype expression matrix. The technique comprises 547 biomarkers that distinguish 29 human immune cell phenotypes, which include T-cells, B-cells, plasma cells, and myeloid subpopulations. The study examined patient data by utilizing the ssGSEA algorithm to determine the relative proportions of 29 immune-infiltrating cells. To more accurately identify immune cell subgroups of interest and validate the results of ssGSEA, we further utilized the CIBERSORT algorithm to identify the relative composition of immune cells in tissues. Samples with *P*-values less than 0.05 were the only ones included in the analysis of immune cell fractions. Additionally, correlation analyses were carried out aiming to investigate the relevance between gene expression and immune cell proportions.

We also utilized the TISIDB (http://cis.hku.hk/TISIDB/index.php), an integrated repository portal for immune system interactions, to explore the correlation between the clusters and five categories of immune regulatory genes, namely receptor-related genes, chemokine-related genes, immunoinhibitor-related genes, MHC-related genes, and immunostimulator-related genes.

### Gene set variation analysis (GSVA)

Gene set variation analysis (GSVA) is an unsupervised and non-parametric technique utilized to evaluate gene set enrichment in transcriptomes. GSVA reallocates gene-level modifications of genes to pathway-level modifications of pathways by integrating the scoring of gene sets of interest, to establish the biological functions of the samples. In this study, we retrieve gene collections from the Molecular Signatures Database (MsigDB) version 7.0 and apply the GSVA algorithm to assess each collection comprehensively, to examine potential alterations in the biological functions of diverse samples.

### Gene set enrichment analysis (GSEA)

The study investigated the signaling pathway discrepancies between groupings with high and low expression through GSEA. The gene set used for background information was obtained from the annotated gene set of the MsigDB, which served as the annotation gene set for the cluster pathways. This gene set was utilized for the differential expression analysis of pathways between clusters. The enriched gene sets (adjusted *P*-value less than 0.05) were then ranked based on their concordance score. The GSEA analysis is commonly employed to investigate disease classification and biological significance.

### Immunofluorescence staining for co-localization validation

To observe the co-localization between hub genes and ECs. Four samples per group of CCMs and normal control samples were collected from the Huashan hospital cohort (HS-cohort). The sections of samples were fixed in 10% formalin and paraffin-embedded, then underwent deparaffinization and antigen retrieval. After blocking at room temperature for an hour, the sections were then incubated overnight at 4°C with the following primary antibodies: BTBD10 (Broad-complex, tram-track and bric-a-brac domain 10) (1:200, sc-377183, Santa Cruz Biotechnology), CD144 (VE-cadherin, 1:200, 14-1441-82, Thermo Fisher Scientific), KIAA1199 (CEMIP (Cell migration-inducing protein), 1:200, DF12056, Affinity Biosciences), and PFDN4 (Prefoldin 4) (1:200, 16045-1-AP, ProteinTech). Subsequently, were then washed three times with PBS for 10 minutes each time and subsequently reacted with Alexa Fluor 488 (1:1000, ab150081, Abcam), or Alexa Flour 594 (1:1000, ab150120, Abcam) secondary antibodies for 1 hour at room temperature, and washed three times with PBS. Coverslips were mounted on slides using anti-quencher medium (P0128, Beyotime). Pannoramic SCAN (3DHISTECH, Hungary) was used for imaging. immunofluorescence images were captured with identical exposure settings. An exposure time series experiment was on CCM samples to determine the optimal exposure time. During this process, the exposure time was progressively adjusted until the desired signal intensity was attained, avoiding overexposure or signal saturation. Subsequently, a fixed exposure time was chosen and applied to all samples. The scanning parameters, including resolution, exposure time, gain, and contrast, were standardized to mitigate potential image bias resulting from parameter variations.

### Western blot for expression validation

Four CCMs samples and normal four control tissues were collected from the HS-cohort for total protein extraction. Protein concentration was measured using the BCA assay, and sample amounts were adjusted to the same total protein level. SDS-PAGE was performed, followed by membrane transfer. The membrane was blocked with 5% BSA or non-fat milk and then incubated overnight at 4°C with the primary antibody: BTBD10 (1:1000, sc-377183, Santa Cruz Biotechnology), KIAA1199 (CEMIP, 1:500, DF12056, Affinity Biosciences), PFDN4 (1:1000, 16045-1-AP, ProteinTech) and β-actin (1:1000, ab8226, Abcam). Afterward, the membrane was incubated with the secondary antibody (ab205718 or ab205719, Abcam) at a 1:2000 dilution for 1-2 hours at room temperature, followed by ECL luminescence development. Grayscale values were analyzed, and protein levels were expressed as a ratio to the internal reference.

### Single-cell RNA-seq analysis

#### Database, preprocessing and integration

We processed a total of 32,261 cells sourced from the GEO database under accession number GSE155788, which were originated from two CCM3^WT^ mice (controls) and two CCM3^KO^ mice (CCM model). Cell capture and library preparation were performed using the 10× Genomics Chromium System and Single Cell 3’ Reagent Kits v2. Sequencing was conducted on an Illumina NovaSeq 6000 system.

Seurat (V5.0.3) was used to perform downstream analysis. Initially, genes expressed in fewer than 10 cells were excluded. To ensure exclusion of potentially damaged cells, cells have < 200 unique molecular identifiers (UMIs) or more than 5 median absolute deviations of the population, as well as cells with over 5% mitochondrial gene expression were dropped. Additionally, potential doublets were identified using scDblFinder (V1.16) and excluded, allowing altogether 28,601 cells for further analysis.

Gene expression of these cells was then log normalized to a maximum of 10,000 and scaled, with number of total UMIs regressed using the ‘ScaleData’ function. We next identified highly variable genes and then performed principal component analysis (PCA) to reduce dimensionality. Top 30 PCs were used to find anchors in canonical correlation analysis (CCA) using the ‘IntegrateLayers’ function, which generated the integrated database of all individual samples.

#### Clustering and annotation

The integrated database was used to identify clusters under the resolution of 0.5 selected based on clustree (V0.5.1). For each identified cluster, marker genes that were conserved across genotypes were identified using the ‘FindMarkers’ function. And then, clusters were annotated based on canonical maker genes of endothelial subtypes previously published [40]. Macrophage/Microglia (Mφ/MG) based were subdivided based on the ratio of M1 marker genes (Tnf, Il1b, Il6, Cd80, Cd68, Cd86, Fcgr1, Nos1, Cybb) and M2 marker gene (Tgfb1, Ccl17, Mrc1, Chil3, Fcgr2b, Pparg) expression. The mean M1/M2 ratio in all Mφ/MG were established as threshold.

#### Cell-cell interaction inference

To analyze intercellular communication within our integrated cell database, CellChat (V1.6.1) [41]was employed (default parameters) to infer potential signaling interactions between cells based on a predefined database of ligand-receptor pairs. Special attention was given to the interactions between various endothelial subtypes and immune cells.

### Statistical analysis

All statistical analysis were performed utilizing R software 4.2.2. Student’s t-test or Wilcoxon were utilized to investigate the difference between two groups. Correlations between variables were assessed using Pearson or Spearman parameters. Differences in the mean were declared statistically significant if *P* < 0.05 and the following statistical significance indicators are used: ^*^P < 0.05; ^**^ *P* < 0.01; ^***^ *P* < 0.001; ^****^ *P* < 0.0001.

## Results

### Feature and functional alteration in CCMs

**Fig. 2:**
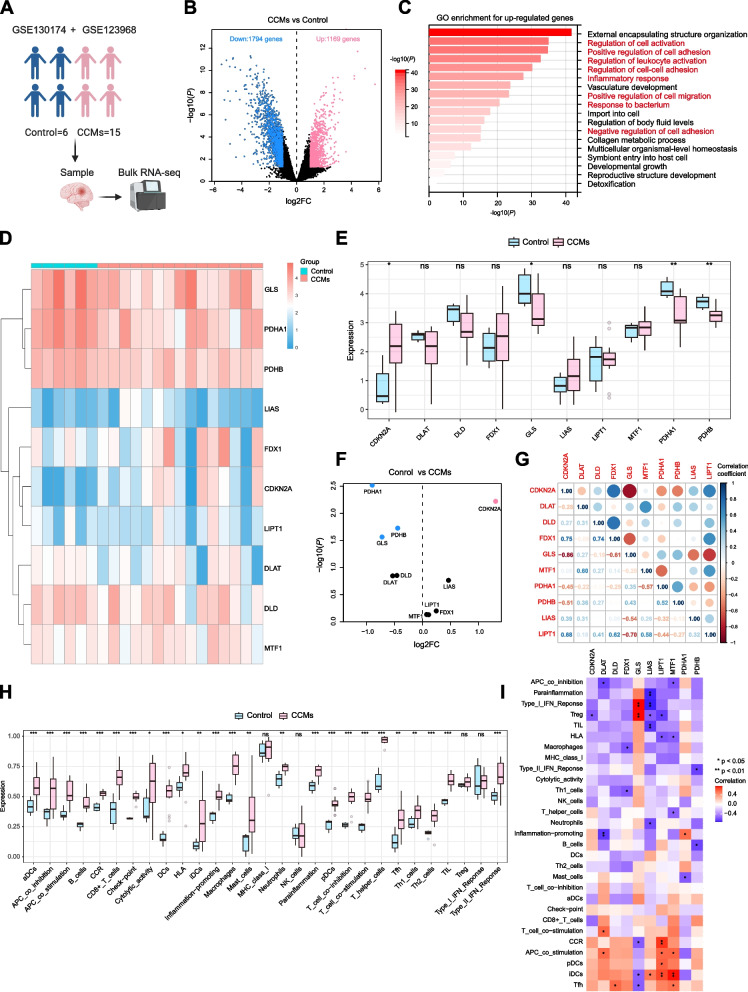
CRGs expressed in control and CCMs group. **A** The GEO datasets and number of samples were illustrated in the schematic. Created with Biorender.com. **B** Volcano plot showing up/down-regulated DEGs of the CCMs *vs.* Control group. Blue dots represent down-regulated DEGs while red dots represent up-regulated DEGs. **C** Bar chart showing GO enrichment for up-regulated genes. The value of -log10(*P*) is represented by the depth of red color and immunity related GO terms are marked in red. **D** The expression patterns of 10 CRGs were presented in the heatmap. The color bar on the right indicates relative expression levels of CRGs (blue: low expression level; red: high expression level). **E** Box plots of CRGs in control group compared to CCMs group. Values are represented as the mean ± SD. **F** Volcano plot of CRGs for Control *vs.* CCMs. Blue dots represent low expression, while red dots represent high expression. **G** Heatmap of correlation of CRGs. The color bar indicates the Pearson correlation coefficient. Blue represents positive correlation, red represents negative correlation and the depth of the color represents the strength of the correlation, and abs (correlation) was displayed by the size of the dot. **H** Box plots showed the difference of immune infiltration assessed with ssGSEA in control group compared to CCMs group. Values are represented as the mean ± SD. **I** The correlation analysis between 10 CRGs and immune cells. The color bar indicates the Pearson correlation coefficient. Red represents positive correlation, purple represents negative correlation, and the depth of the color represents the strength of the correlation. *P-*values in the box plots are denoted by asterisks: ^*^P-<0.05, ^**^P<0.01, ^***^P<0.001 and ns indicates no significant difference. CRGs, Cuproptosis-related genes; CCMs, Cerebral cavernous malformations; DEGs, Differentially expressed genes; GO, Gene Ontology; ssGSEA, Single-sample Gene Set Enrichment Analysis

We analysed an expression profiling data obtained from 21 patients, which comprises a control group of six patients and a CCMs group of 15 patients (Fig. 2A). We found a total of 2963 DEGs between CCMs and control groups, with 1169 genes upregulated and 1794 genes downregulated in CCMs (Fig. 2B). Subsequent GO enrichment analysis found that the upregulated genes were mainly enriched in GO terms associated with immunity and vascular generation (Fig. 2C).

### CRGs expression and immune infiltration analysis in CCMs

To further investigate the expression patterns of CRGs in CCMs, CRGs were sourced from a previous publication [42], and the subsequent list was limited to those genes with human bulk RNA expression data available in GSE130174 and GSE123968. Our findings suggested that four cuproptosis genes, namely CDKN2A, GLS, PDHA1, and PDHB exhibited significant expression differences between the control and CCMs group (Fig. 2D-F). Specifically, CDKN2A was highly expressed in CCMs, while GLS, PDHA1, and PDHB were highly expressed in the control group. It's worth noting that FDX1 and LIAS showed increased expression in CCMs compared to the control group, although there was no significant difference (Fig. 2E, F). Additionally, we conducted correlation for CRGs to probe CRGs’role in CCMs development. Notably, FDX1 was positively correlated with CDKN2A and DLD. In contrast, GLS was negatively correlated with CDKN2A and FDX1. LIAS was negatively correlated with GLS. LIPT1 was positively correlated with CDKN2A, FDX1, and MTF1, and negatively correlated with GLS. Moreover, CDKN2A was positively correlated with PDHA1 and PDHB (Fig. 2G). Given that upregulated DEGs in CCMs are primarily enriched in immune regulatory in CCMs (Fig. 2C), we performed CIBERSORT analysis to assess the immune infiltration of the samples. Our analysis revealed significant immune cell infiltration in CCMs compared to the control group, including macrophages, dendritic cells (DCs), B cells, and T cells (Fig. 2H). The analysis of the relationship between CRGs and immune cells showed that most CRGs were positively correlated with CCR (C-C chemokine receptor), T cell co-stimulation, APC (Antigen Presenting Cell) co-stimulation, DCs and Tfh (T follicular helper cell). In contrast, they displayed negative correlations with APC co-inhibition, parainflammation, Treg (Regulatory T cell), Type I IFN Response, TIL (Tumor-Infiltrating Lymphocyte), HLA (Human Leukocyte Antigen) and macrophages (Fig. 2I).

### Consensus clustering CCMs based on expression of CRGs

**Fig. 3:**
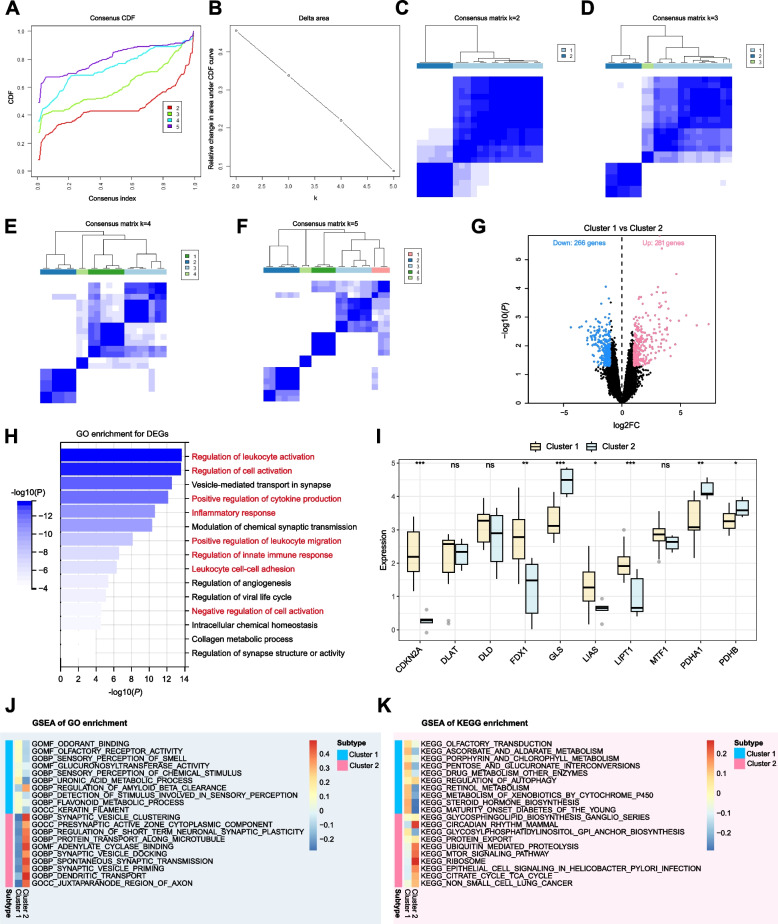
Identification of cuproptosis-related molecular subtypes and comprehensive pathway enrichment analysis in CCMs. **A** CDF curves displayed consensus distributions from k=2 to k=5. **B** Area fraction under the CDF curve for k =2–9. The horizontal axis indicated the number of categories (k), while the vertical axis indicated the relative changes in the area under the CDF curves. **C** - **F** Consensus clustering matrixes were generated for values of k ranging from 2 to 5. **G** Volcano plot showing up/down-regulated DEGs of the Cluster 1 *vs.* Cluster 2. Blue dots represent down-regulated DEGs while red dots represent up-regulated DEGs. **H** Bar chart showing GO enrichment for DEGs of Cluster 1 *vs.* Cluster 2. The value of -log10(*P*) is represented by the depth of blue color and immunity related GO terms are marked in red. **I** Box plot visualized the expression patterns of CRGs in two CCMs clusters. Values are represented as the mean ± SD. GESA of (**J**) GO enrichment and (**K**) KEGG enrichment of with bar plots based on the expression level of cluster genes. The color bar on the right indicates gene expression levels. The gene sets of each cluster were subdivided into high-expression and low-expression groups, with red color representing the high-expression genes from the respective cluster, and dark indicating the low-expression genes from the corresponding cluster, respectively. *P*-values in the box plot are denoted by asterisks: ^*^P < 0.05, ^**^P < 0.01, ^***^P < 0.001 and ns indicates no significant difference. CDF, Cumulative distribution function; KEGG, Kyoto Encyclopedia of Genes and Genomes; GSEA, Gene Set Enrichment Analysis

We employed a consensus clustering approach to investigate the pattern of modifications in CCMs cuproptosis based on assessing expression discrepancy of CRGs. The result of analysis revealed a clearer distinction between the two sample clusters at k=2, resulting in the establishment of two distinct clusters (Fig. 3A-F). Volcano plot was plotted to depict the distribution of DEGs between two clusters, consisting of 281 upregulated genes and 266 downregulated genes when Cluster 1 *vs.* Cluster 2 (Fig. 3G). The GO enrichment analysis revealed that the DEGs were predominantly enriched in immune regulation, vascular development, and synapses (Fig. 3H). We further investigated the expression of CRGs within these clusters and identified seven genes displaying differential expression (Fig. 3I). Specifically, CDKN2A, FDX1, GLS, LIAS, LIPT1, PDHA1, and PDHB were found to exhibit differential expression patterns between the two clusters. CDKN2A, FDX1, LIAS, and LIPT1 had high expression levels in cluster 1, while GLS, PDHA1, and PDHB had high expression levels in Cluster 2. As FDX1 and LIAS play a significant positive regulatory role in cuproptosis-induced cell death, while GLS plays a negative regulatory role [22], patients in Cluster 1 featuring a high expression of FDX1 and LIAS might be more prone to experiencing a positive promotion of cuproptosis, whereas patients in Cluster 2 exhibiting a high expression of GLS could obtain the opposite regulatory effect.

### Functional enrichment between clusters

To explore the functions of genes from two clusters within the samples, GSEA enrichment analysis was conducted to identify the enriched gene sets for each cluster. The 20 most significant pathways for Cluster 1 and Cluster 2 were chosen for heatmapping, which highlighted divergent pathways enriched in each isoform (Fig. 3J, K). Notably, in the GSEA of GO enrichment, GLUCURONOSYLTRANSFERASE ACTIVITY is significantly activated in the gene high-expression group of Cluster 1, while ADENYLATE CYCLASE BINDING is activated in the gene high-expression group of Cluster 2 (Fig. 3J). In the GSEA of KEGG enrichment, ASCORBATE AND ALDARATE METABOLISM, ITOSE AND GLUCURONATE INTERCONVERSIONS, and METABOLISM OF XENOBIOTICS BY CYTOCHROME P450 are mainly enriched in the gene high-expression group of Cluster 1, while GLYCOSYLPHOSPHATIDYLINOSITOL GPI ANCHOR BIOSYNTHESIS and CITRATE CYCLE TCA CYCLE are enriched in the gene high-expression group of Cluster 2 (Fig. 3K). It is worth noting that these pathways involving mitochondrial energy transfer and metabolism may be involved in regulating cuproptosis, which primarily occurs in the mitochondria.

### Hub genes associated with different subtypes of CCMs

**Fig. 4:**
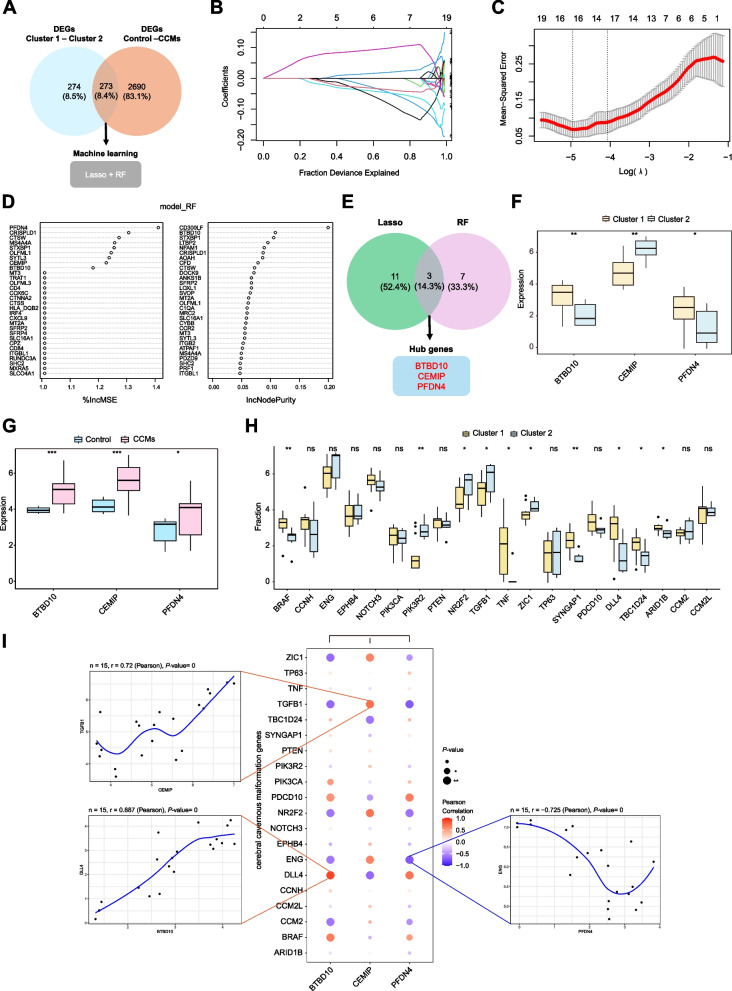
Identification of CRGs associated hub genes in two CCMs clusters. **A** Venn plot displaying 273 overlapping DEGs of Cluster 1 *vs.* Cluster 2 and Control *vs.* CCMs. The overlapping genes were subsequently analyzed using the LASSO regression algorithm and the random forest algorithm. **B** DEGs profiles based on LASSO coefficients. **C** LASSO coefficient values of the DEGs. The vertical dashed lines are the optimal log(λ) values. **D** DEGs profiles based on random forest algorithm. **E** Venn plot displaying 3 overlapping genes selected by LASSO regression algorithm and random forest algorithm. **F** Box plots compared the expression of BTBD10, CEMIP, PFDN4 in two CCMs clusters. **G** Box plots compared the expression of BTBD10, CEMIP, PFDN4 in control and CCMs group. **H** The comparisons of the expression of CCMs-related genes between two CCMs clusters. Values are represented as the mean ± SD. (**I**) Bubble map for the Pearson correlations between three hub genes (BTBD10, CEMIP, PFDN4) and CCMs-related genes. *P*-value was represented by the size of circle. The bigger the circle, the closer the *P*-value was to zero; Pearson correlation coefficient was represented by color. The redder the color, the stronger the positive correlation; The deeper of the purple color, the stronger the negative correlation. *P*-values in the box plots are denoted by asterisks: ^*^P < 0.05, ^**^P < 0.01, ^***^P < 0.001 and ns indicates no significant difference. LASSO, least absolute shrinkage and selection operator; RF, random forest. BTBD10, Broad-complex, tram-track and bric-a-brac domain 10; CEMIP, Cell migration-inducing protein; PFDN4, Prefoldin 4; DLL4, Delta-Like Canonical Notch Ligand 4; TGFB1, Transforming Growth Factor Beta 1; ENG, endoglin

We further meticulously selected 273 intersecting DEGs from Cluster 1 *vs.* Cluster 2 and Control *vs.* CCMs for screening feature hub genes associated with different subtypes of CCMs clustered by CRGs (Fig. 4A). The LASSO regression and random forest algorithms were employed in the screening process. There are 14 feature genes that are related to CCMs identified through LASSO regression analysis, as shown in Fig. 4B and C. The Random Forest algorithm selected a set of 10 feature genes (Fig. 4D), which were then intersected with the feature genes obtained from the LASSO regression algorithm to derive a final list of three overlapping genes, namely PFDN4, CEMIP, and BTBD10 (Fig. 4E), that were investigated as the hub genes in our upcoming study. Upon comparing the two clusters, we observed that BTBD10 and PFDN4 were highly expressed in Cluster 1, while CEMIP was highly expressed in Cluster 2. Furthermore, all three genes were significantly overexpressed in CCMs (Fig. 4F and G).

Pathogenic genes related to CCMs were identified using the GeneCards database. Varying expression levels of known pathogenic genes among clusters were demonstrated in Fig. 4H. B-Raf proto-oncogene, serine/threonine kinase (BRAF), Tumor Necrosis Factor (TNF), Synaptic Ras GTPase Activating Protein 1 (SYNGAP1), Delta-Like Canonical Notch Ligand 4 (DLL4) and AT-Rich Interaction Domain 1B (ARID1B) were highly expressed in Cluster 1; while Phosphoinositide-3-Kinase Regulatory Subunit 2 (PIK3R2), Nuclear Receptor Subfamily 2 Group F Member 2 (NR2F2), Transforming Growth Factor Beta 1 (TGFB1), and Zic Family Member 1 (ZIC1) were highly expressed in Cluster 2. Furthermore, the expression levels of three hub genes displayed a significant correlation with various disease-associated genes. This includes a remarkable positive correlation between BTBD10 and DLL4 (r = 0.887, *P*<0.001) and a significant negative correlation between PFDN4 and endoglin (ENG) (r = -0.725, *P*<0.001) (Fig. 4I). Additionally, CEMIP exhibited a positive correlation with TGFB1 (r = 0.75, P<0.001). It is noteworthy that the expression patterns of BTBD10 and PFDN4 are generally consistent with CCMs pathogenic genes, while being opposite to CEMIP.

### Immune infiltration characteristics of the CCMs subtypes

**Fig. 5:**
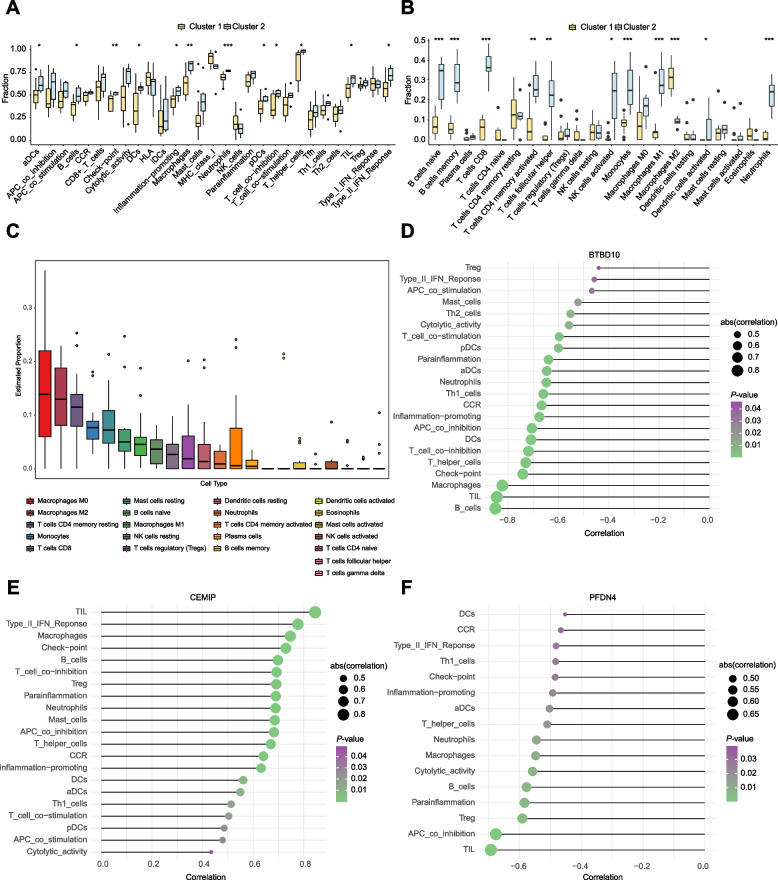
Analysis of immune infiltration for hub genes from two CCMs clusters. **A** Box plot showed the difference of immune infiltration assessed with ssGSEA in Cluster 1 compared to Cluster 2 group. **B** Box plot showed the difference of immune cell subtypes validated by CIBERSORT algorithm in Cluster 1 compared to Cluster 2 group. **C** Box plot showed the estimated proportion of immune cells from two clusters assessed by CIBERSORT algorithm. Immune cells were annotated by using different colours. Values are represented as the mean ± SD. *P*-values in (**A**) and (**B**) are denoted by asterisks: ^*^P < 0.05, ^**^P < 0.01, ^***^P < 0.001. **D-F** Bubble map for the correlations between hub genes from two CCMs clusters and immune cells. Circles on the right indicates the absolute value of the correlation coefficient. The bigger the circle, the stronger the positive/negative correlation. *P*-value was indicated by color. The deeper of the green color, the closer the *P*-value was to zero

The immune microenvironment is composed mainly of immune cells, extracellular matrix, various growth, and inflammatory factors, as well as distinctive physicochemical features. These elements significantly affect the CMMs development or hemorrhage. We conducted an primary analysis by ssGSEA to investigate the potential molecular mechanisms that contribute to the progression of CCMs through examining the relevance between two clusters and immune infiltration. Statistical disparities were observed in a variety of immune cells, including activated dendritic cells (aDCs), B cells, check-point, DCs, inflammation-promoting cells, macrophages, neutrophils, plasmacytoid dendriticcells (pDCs), T cell co-inhibition, T helper cells, TIL, and Type II IFN Response between Cluster 1 and Cluster 2, and Cluster 2 exhibits significantly higher levels of immune infiltration than Cluster 1. (Fig. 5A). The CIBERSORT algorithm was then used to validate the results of ssGSEA, yielding consistent outcomes (Fig. 5B). Notably, macrophages, particularly M0 and M2 types, constitute a significant proportion of all immune cells assess by CIBERSORT (Fig. 5C).

Furthermore, we investigated the correlation between hub genes and immune cells and found that the hub genes BTBD10 and PFDN4 from Cluster 1 exhibited a significant negative correlation with most immune cells, including macrophages and B cells (Fig. 5D and F). In contrast, the hub gene CEMIP from Cluster 2 demonstrated a significant positive correlation with most immune cells (Fig. 5E). The analysis indicated a close association between the hub genes from two clusters and the degree of immune cell infiltration, signifying their significant role in the immune microenvironment in CCMs sbutypes.

### Differential analysis of immune system interactions in the CCMs subtypes

We extracted various immune factors from the TISIDB database and analysed for intergroup variation in different immune factor clusters, including immune-related chemokines, immunosuppressive, and immunostimulatory factors. Our results show a significant upregulation of Chemokine (C-X-C motif) ligand 9 (CXCL9), Chemokine (C-C motif) ligand 22 (CCL22), Chemokine (C-X-C motif) ligand 12 (CXCL12), as well as immunosuppressive factors including Colony-stimulating factor 1 receptor (CSF1R), IL10, TGFB1, TGFBR1, CD274, and Programmed cell death 1 ligand 2 (PDCD1LG2) in Cluster 2 (Fig. S1A-B). Interesting, several immunostimulatory factors were found to significantly increase in Cluster 2, including CD27, CD8, CD86, Ectonucleoside triphosphate diphosphohydrolase-1 (ENTPD1), CD40, 5’-Nucleotidase Ecto (NT5E), and Tumor Necrosis Factor Superfamily Member 13b (TNFSF13B) (Fig. S1C). On the other hand, Cluster 1 showed significant increases in MHC-related genes such as Major Histocompatibility Complex, Class II, DO beta (HLA-DOB), Major Histocompatibility Complex, Class I, G (HLA-G), and Transporter 2, ATP Binding Cassette Subfamily B Member (TAP2) (Fig. S1C). These results indicate that the interactions with the immune system may differ between Cluster 1 and Cluster 2."

### Signaling pathways enrichment of hub genes

**Fig. 6:**
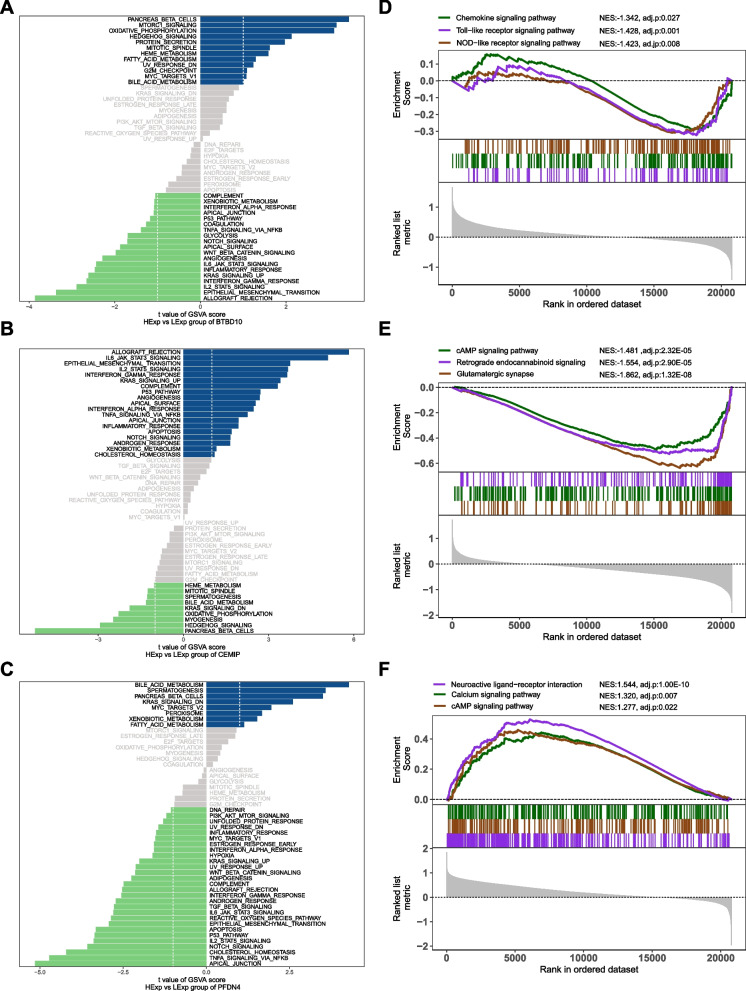
GSVA and GSEA analysis of pathways activated upon up- and down-regulation of BTBD10, CEMIP, PFDN4. **A** GSVA of BTBD10; (**B**) GSVA of CEMIP; (**C**) GSVA of PFDN4. Differences in pathway activities scored by GSVA, between high expression and low expression (from up to down) of specific gene. The GSVA scores, serving as the X-axis, are sorted in descending order according to their rank, to reflect the significant pathways enrichment levels of gene set, and two K-S statistical distribution lines are drawn. Blue and green colors represent significantly enriched pathways, while gray color indicates non-differential pathways. (**D**) GSEA of BTBD10; (**E**) GSEA of CEMIP; (**F**) GSEA of PFDN4. GSVA, Gene Set Variation Analysis; NES, Normalized Enrichment Score; adj.p, adjust *P*-value

We analyzed the signaling pathways enriched by the three hub genes to investigate their potential molecular mechanisms through which they impact the progress of CCMs. The GSVA results indicate that BTBD10, when highly expressed, primarily activates signaling pathways like mTORC1, fatty acid metabolism, hedgehog and PI3K-ATK-MTOR signaling as shown in Fig. 6A, B indicates that elevated expression of CEMIP predominantly enriched in IL6/JAK/STAT3, angiogenesis, TNFA signaling via NFκB and inflammation response signaling pathways. Meanwhile, the significant enrichment of PFDN4 expression was identified in bile acid metabolism, KRAS signaling and fatty acid metabolism as illustrated in Fig. 6C. Furthermore, the results of GSEA analysis demonstrate that BTBD10 is enriched in the pathways of Chemokine, NOD-like receptor, and Toll-like receptor signaling pathways (Fig. 6D); CEMIP is enriched in the cAMP signaling pathway, Glutamatergic synapse, and Retrograde endocannabinoid signaling (Fig. 6E); and PFDN4 is enriched in the Calcium signaling pathway, cAMP signaling pathway, and Neuroactive ligand-receptor interaction (Fig. 6F). These findings suggest that hub genes can significantly impact the progression of CCMs by regulating vascular endothelial growth, inflammatory response, mitochondrial energy metabolism and cell cycle.

### Validation of hub genes in human ECs

**Fig. 7:**
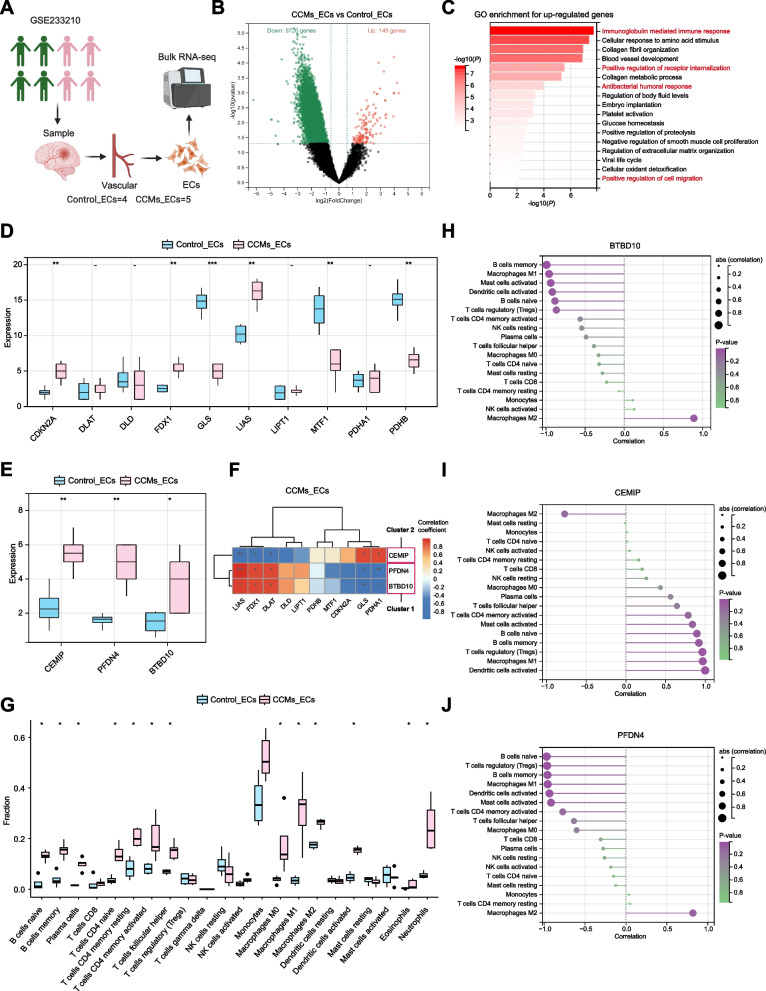
Validation of hub genes using an external bulk RNA-seq dataset from human CCMs ECs. **A** The human CCMs bulk RNA-seq dataset and number of EC samples were demonstrated in the schematic. Created with Biorender.com. **B** Volcano plot showing up/down-regulated DEGs of the CCMs_ECs *vs.* Control_ECs group. Green dots represent down-regulated DEGs while red dots represent up-regulated DEGs. **C** Bar chart showing GO enrichment for up-regulated genes. The value of -log10(*P*) is represented by the depth of red color and immunity related GO terms are marked in red. **D** Box plots of CRGs in control_ECs group compared to CCMs_ECs group. **E** Box plots of hub genes in control_ECs group compared to CCMs_ECs group. **F** Heatmap displays the relationship between hub genes and CRGs in the CCMs_ECs group. The correlation coefficients are represented using color bar, where red indicates a positive correlation and blue indicates a negative correlation. **G** Box plots showed the difference of immune infiltration assessed with CIBERSORT in control_ECs group compared to CCMs_ECs group. Values are represented as the mean ± SD. *P*-values in the heatmap and box plot are denoted by asterisks: ^*^P < 0.05, ^**^P < 0.01, ^***^P < 0.001. **H** - **J** Bubble map for the correlations between hub genes and immune cells. Circles on the right indicates the absolute value of the correlation coefficient. The bigger the circle, the stronger the positive/negative correlation. *P*-value was indicated by color. The deeper of the purple color, the closer the *P*-value was to zero

Considering the relevance of CCMs in the context of angiogenesis, a GEO dataset (GSE233210) that contains bulk RNA-seq data from human ECs was selected to validate the expression and function of hub genes in human ECs (Fig. 7A). In the comparison of CCMs_ECs and Control_ECs, a total of 149 DEGs with upregulation and 9,726 DEGs with downregulation were identified (Fig. 7B). The up-regulated DEGs were found to enriched in GO terms associated with immune regulation and blood vessel development (Fig. 7C), consistent with the results of the primary analysis (Fig. 2C). In term of the expression of CRGs, CDKN2A, FDX1 and LIAS were found to up-regulated in CCMs_ECs group, while GLS, MTF1 and PDHB were found the up-regulated in Control_ECs group (Fig. 7D). Therefore, the CCMs_ECs group appears to exhibit more features related to cuproptosis. Meanwhile, we found that hub genes BTBD10, PFDN4, and CEMIP were significantly upregulated in the CCMs_ECs (Fig. 7E).

We further conducted a correlation analysis between hub genes and CRGs in the CCMs_ECs group. The results showed that the expressions of BTBD10 were consistent with PFDN4 in the analysis of relationship with CRGs, significantly positively correlated with FDX1, LIAS, and DLAT, and significantly negatively correlated with GLS and PDHA1 (Fig. 7F). It is worth mentioning that CEMIP showed the opposite correlation results with BTBD10 and PFDN4, significantly negatively correlated with FDX1, LIAS, and DLAT, and significantly positively correlated with GLS and PDHA1. The results are also highly consistent with the clustering based on CRGs expression in primary analysis (Fig. 3I).

The results of immune infiltration analysis showed that the CCMs_ECs group had a higher infiltration of immune cells (Fig. 7G), consistent with the primary analysis results of CCMs gross specimens (Fig. 2H). The correlation analysis between hub genes and immune cells revealed that BTBD10 and PFDN4 were negatively correlated with most immune cells, while CEMIP was positively correlated with immune cells (Fig. 7H-J). Interestingly, BTBD10 and PFDN4 were positively correlated with M2 macrophages but negatively correlated with M1 macrophages (Fig. 7H and J), while CEMIP showed the opposite correlation pattern (Fig. 7I).

### Validation of hub genes in HS-cohort

**Fig. 8:**
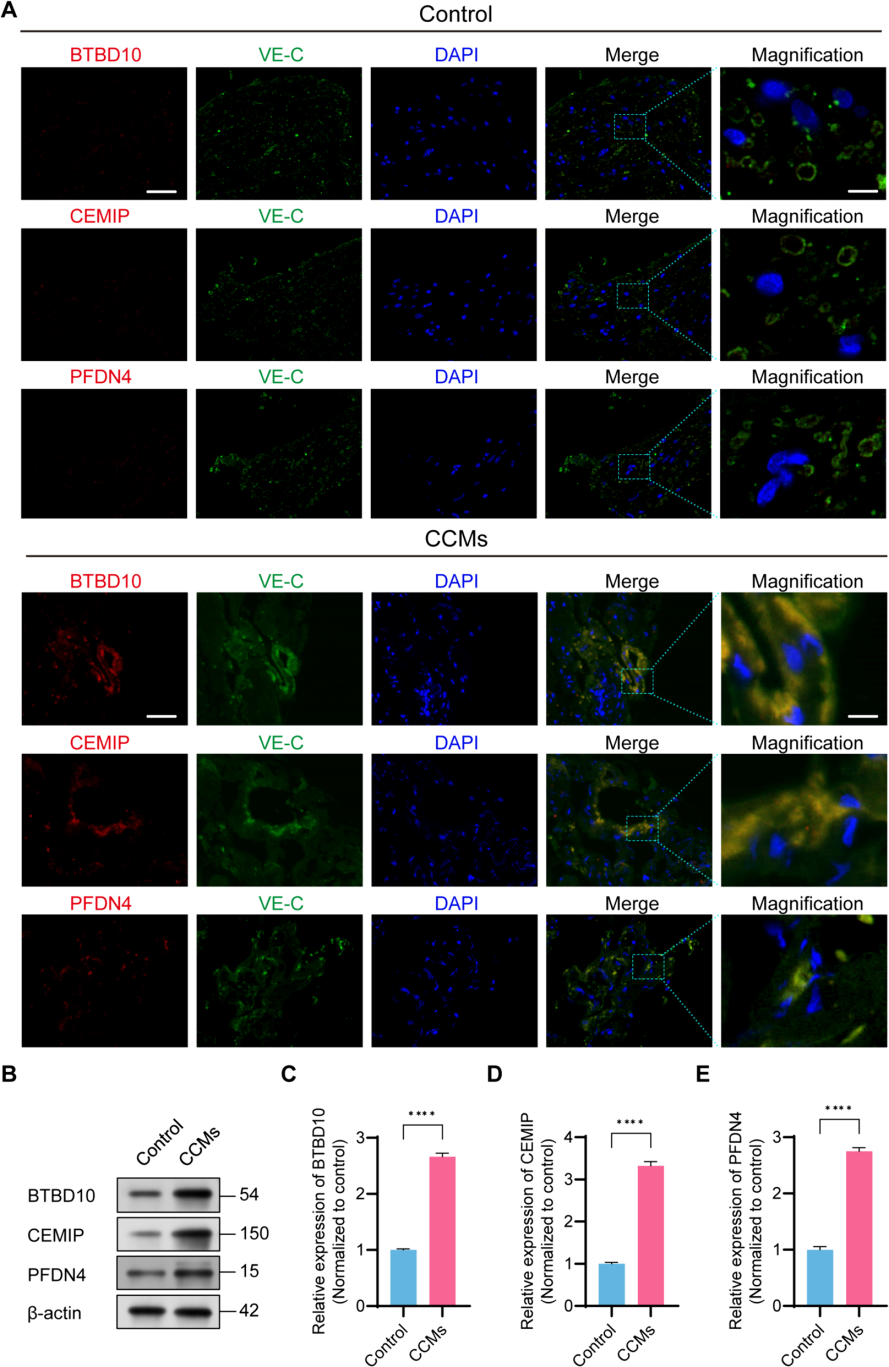
Expression of hub genes were validated between control and CCMs group in HS-cohort. **A** Representative Immunofluorescence images for co-localization of BTBD10, CEMIP, PFDN4 with VE-C, scale bar = 50 µm for low magnification; scale bar = 10 µm for high magnification; (**B-E**) Western blotting and quantitative analysis of BTBD10, CEMIP, PFDN4. N=4 for each group. Values are represented as the mean ± SD. *P-*values are denoted by asterisks: ^*^P< 0.05, **P< 0.01, ^***^P< 0.001 and ns indicates no significant difference. VE-C, VE-cadherin. HS, Huashan hospital

To further ascertain the actual expression of these three hub genes in CCMs, we performed immunofluorescence co-localization and assessed protein expression levels in CCMs sample collected from HS-cohort. We observed a co-localization of BTBD10, CEMIP, and PFDN4 with VE-cadherin (a distinctive marker of ECs) in the tissues of CCMs (Fig. 8A). Moreover, our results revealed that the levels of BTBD10, CEMIP, and PFDN4 were significantly elevated in the CCMs group compared to the control group, which signified functional expression of these hub genes in the CCMs tissues (Fig. 8B-E).

### Validation of hub genes at the single cell level

**Fig. 9:**
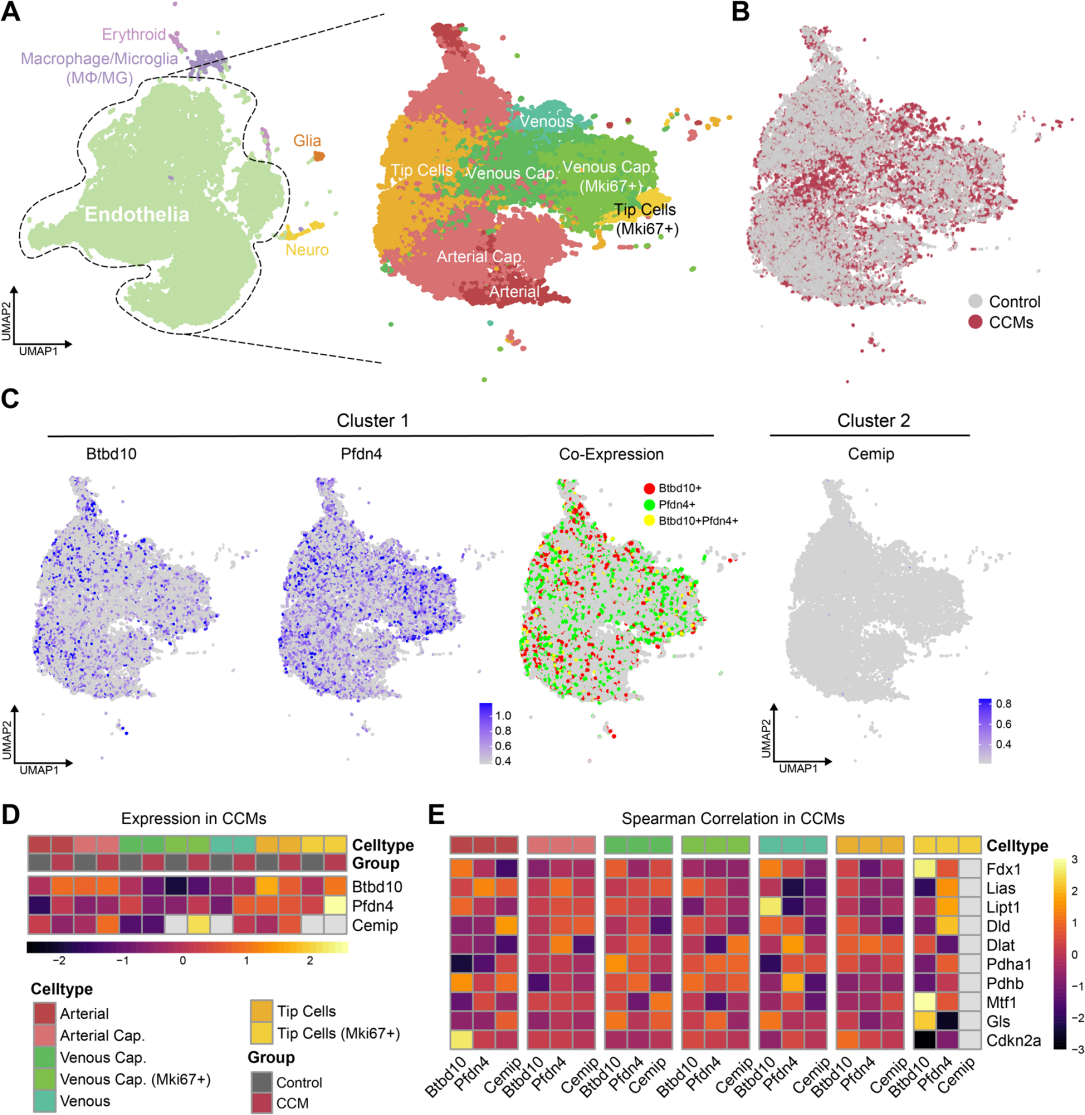
scRNA-seq analysis delineates Mki67+ Tip cells as key endothelial subclusters involved in cuproptosis in CCMs mouse models. **A** UMAP plots depict 5 major cell types (left) and 6 endothelial subtypes (right) as identified by Leiden clustering, with each dot representing an individual cell color-coded by type or subtype; **(B**) A UMAP plot contrasts the cellular distribution between Control (CCM3^WT^) and CCM (CCM3^KO^) groups; (**C**) UMAP plot illustrates the expression patterns of cuproptosis hub genes (Btbd10, Pdnf4) and their co-expression in Cluster 1, alongside Cemip in Cluster 2, with gene expression levels normalized for projection; (**D**) Heatmap represents the correlation between hub genes (Btbd10, Pdnf4, Cemip) and CRGs (Fdx1, Lias, Lipt1, Dld, Dlat, Pdha1, Pdhb, Mtf1, Gls, Cdkn2a) across endothelial subclusters in CCMs, with Spearman’s rank correlation coefficients presented as z-scores; (**E**) Heatmaps display the expression levels of hub genes across endothelial subclusters within Control and CCMs groups, with color gradients indicating z-scored expression levels

A total of four samples (two normal and two CCMs samples) were obtained for analysis in this study, which came from scRNA-seq dataset (GSE155788). Among them, 14,623 single cells derived from normal tissues, whereas 15,978 cells were CCMs-derived. The cells were classified into five main cell types, including erythroid, MΦ/MG, glia, endothelia and neuro, and each major cell type was well mixed according to sample classification (Fig. 9A). Based on well-known markers, endothelia were selected to subdivide the cell subgroups, and a total of seven endothelial clusters were identified, including artery, arterial capillary, venous capillary, venous capillary (Mki67+), vein, tip cells, and tip cells (Mki67+) (Fig. 9B and Fig. S2). Fig 9C shows the differences in cell distribution between the control and CCMs groups. These findings indicate the heterogeneous landscape among normal and CCMs samples. Subsequent co-expression analysis showed that Btbd10 and Pfdn4 from Cluster 1 overlapped in multiple ECs in CCMs (Fig. 9C), demonstrating their high-level co-expression at the cellular level. However, we found that Cemip, which originated from Cluster 2, had a relatively low overall expression level in the cell population of CCMs. Further subgroup analysis revealed that Btbd10 and Pfdn4 were highly expressed in tip cells (Mki67+) compared to the control group (Fig. 9D). Moreover, Btbd10 was highly expressed in the artery, while Pfdn4 was highly expressed in vein, venous capillary (Mki67+), and arterial capillary. Cemip was highly expressed in venous capillary (Mki67+), tip cells, and arterial capillary, especially in the venous system.

We subsequently conducted correlation analyses of hub genes and CRGs in different cell subgroups of CCMs, and the results indicated that the relationship between these three hub genes and CRGs was heterogeneous (Fig. 9E). We found that Btbd10 was significantly correlated with multiple CRGs in several cell subclusters, especially in tip cells (Mki67+) where it was positively correlated with three genes, Fdx1, Mtf1, and Gls. Additionally, Btbd10 was positively correlated with Lipt1 in the vein and Cdkn2a in the artery, Therefore, the role of Btbd10 in ECs became the focus of subsequent exploration in this research.

### Potential cuproptosis mechanisms of CCMs regulated by Btbd10 though cellchat between M2 macrophage and tip cells (Mki67+)

**Fig. 10:**
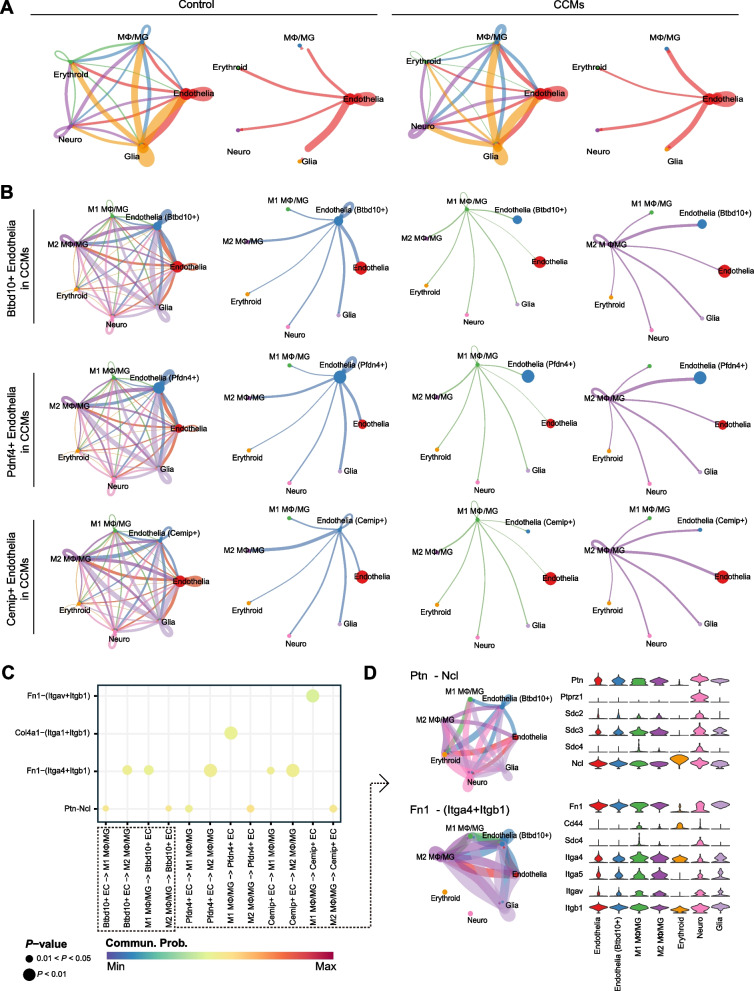
Cell-cell communication dynamics between Mφ/MG and endothelial subclusters expressing identified hub genes in CCMs. **A** Circle plots map the interaction weights among the 5 major cell subclusters in control and CCMs group, color-coded by source cell cluster identity; (**B**) Circle plots details the interaction strengths between endothelial cell subclusters expressing Btbd10, Pdnf4, and Cemip, and other cell subclusters in CCMs, with a focus on M1 and M2 Mφ/MG interactions; (**C**) A dotplot highlights the primary signaling pathways between endothelial and Mφ/MG cells in CCMs, where each dot represents a ligand-receptor pair, sized by pathway involvement *P*-value, and colored by communication probability; (**D**) An illustration of Ptn and Fn1 signaling pathways, including a circle plot (left) showing interaction strengths and a violin plot (right) depicting ligand and receptor gene expression levels

We next aimed to investigate whether the heightened expression of Btbd10 in endothelia coincided with altered intercellular communication in CCMs. For this purpose, we used CellChat [41], a tool that utilizes a database of ligand-receptor interactions to analyze cell-cell communication from scRNA-seq data. Our findings showed significant differences in cell-cell interactions between the control and CCMs samples (Fig. 10A). Specifically, we found that the cell-cell interaction strength between MΦ/MG and most other cells was significantly enhanced in large cell populations, particularly between MΦ/MG and endothelia, which was consistent with our observations in the bulk RNA-seq dataset (Fig. 2H).

Subsequently, we divided MΦ/MG into two subclusters, M1 and M2, to explore their interactions with the high hub gene-expressing endothelial the CCMs samples (Fig. 10B). Our results showed that the strength of interactions between M2 MΦ/MG and Btbd10+ endothelial and Pfdn4+ endothelial were stronger than that of other cells, indicating that these two subclusters of endothelial may play a primary functional role in the interaction with M2 MΦ/MG. This finding was consistent with our observations in the bulk RNA-seq data (Fig. 5D and F). However, the interaction between M2 MΦ/MG and Cemip+ endothelia was weaker compared to that with the overall endothelia, indicating that the interaction between Cemip+ endothelia and M2 MΦ/MG may not be the primary functional interaction.

We further undertook an in-depth analysis of intercellular ligand pathways (Fig. S3), unearthing significant probability of communication between endothelia with highly expressed hub gens and MΦ/MG mainly through pleiotrophin (Ptn) binding to nucleolin (Ncl) and fibronectin (Fn1) binding to integrin alpha 4 (Itga4) and integrin a1 (Itgb1) (Fig. 10C). With the positive association between Bdbt10 and several endothelial subgroups' CRGs taken into account (Fig. 9E), we then explored the notably significant interactive paths between Btbd10+ endothelia and MΦ /MG (Fig. 10D, left panels). Interestingly, we discovered that the primary pathways for connections from M2 MΦ/MG extending to Btbd10+ endothelia were through the Ptn-Ncl pathway. Contrastingly, the connections from Btbd10+ endothelia extending to M2 MΦ/MG were primarily through the Fn1-(Itga4+Itgb1) pathway. Concurrently, we noticed the expression of the corresponding signal pathways' receptors and ligands increase in the corresponding interaction pairs between MΦ/MG and Btbd10+ endothelia (Fig. 10D, right panels).

**Fig. 11:**
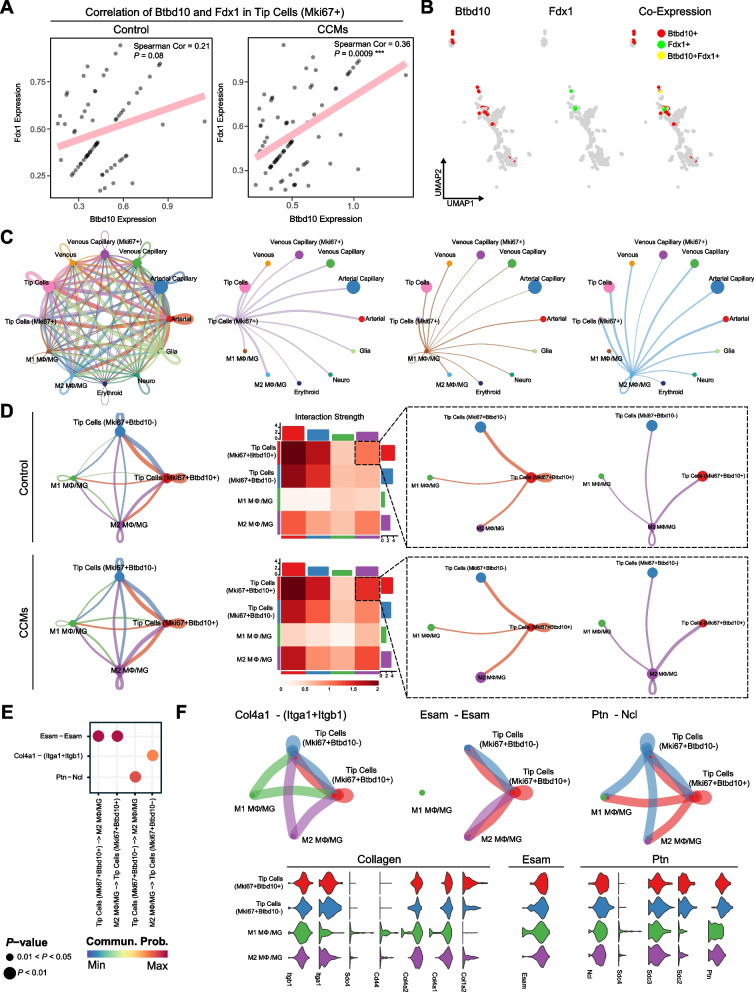
Btbd10 expression in Mki67+ Tip cells is suggested to enhance endothelial-Mφ/MG interaction in CCMs. **A** A scatter plot examines the expression correlation between Btbd10 and Fdx1 in Mki67+ Tip cells, with a linear regression line and Spearman correlation coefficient and *P*-value indicated; (**B**) A UMAP plot visualizes the co-expression of Btbd10 and Fdx1 in Mki67+ Tip cells, projecting normalized gene expression levels; (**C**) Circle plots assesse the interaction strength between endothelial subtypes and Mφ/MG in CCM, focusing on Mki67+ Tip cells; (**D**) Circle plots and heatmap explore the interaction strength between Btbd10-expressing Mki67+ Tip cells and Mφ/MG, differentiated by Btbd10 expression levels, with color gradients reflecting scaled interaction strengths; (**E**) A dotplot identifies major signaling pathways between Btbd10+Mki67+ Tip cells and Mφ/MG in CCMs, using dots to represent ligand-receptor pairs, sized by *P*-value, and colored by communication probability; (**F**) An illustration of Collagen, Esam, and Ptn signaling pathways, featuring a circle plot (top) for interaction strengths and a violin plot (bottom) for gene expression levels

Due to the high expression of Btbd10 in tip cells (Mki67+) (Fig. 9D), we sought to explore and validate the correlation between Btbd10 and cuproptosis in relevant cell subclusters of endothelia. We subsequently analyzed the relationship between Btbd10 and a feature gene of cuproptosis- Fdx1 (Fig. 11A). The results showed that Btbd10 has a substantial positive correlation with Fdx1 in the tip Cells (Mki67+) of CCMs samples, while no significant correlation in the control group. Further colocalization results also hinted at Btbd10 and Fdx1's coexpression in the tip Cell (Mki67+) population (Fig. 11B). These results imply a substantial correlation between Btbd10 and cuproptosis in CCMs at the cellular level.

We moved forward by sorting out tip cells (Mki67+) in endothelia from CCMs samples evaluated their interactions with other cell clusters by using Cellchat analysis. We found that tip cells (Mki67+) have significant interaction strength with MΦ/MG, especially M2 MΦ/MG (Fig. 11C). Simultaneously, compared to tip cells (Mki67+) exhibiting low Btbd10 expression, a significant increase in interaction strength was observed between tip cells (Mki67+) with high Btbd10 expression and M2 MΦ/MG. (Fig. 11D).

Lastly, we analyzed the ligand-receptor pathways between M2 MΦ/MG and tip cells (Mki67+) with high or low expression of Bdbt10 (Fig. S4), finding that they primarily interact through Esam (cndothelial cell-selective adhesion molecule)-Esam, Col4a1-(Itga1+Itgb1) (collagen type IV alpha1 chain-(interacting with integrin a1) +Itgb1), and Ptn-Ncl ligand pathways (Fig. 11E). Among these, the interaction between M2 MΦ/MG and tip cells (Mki67+Btbd10+) primarily takes place via the Esam-Esam ligand pathway. In contrast, the interaction between M2 MΦ/MG and Tip Cells (Mki67+Btbd10-) primarily takes place via Col4a1-(Itga1+Itgb1) and Ptb-Ncl (Fig. 11F). The expression of ligands and receptors in the respective pathways increases in the corresponding interaction pairs between M2 MΦ/MG and tip cells (Mki67+Btbd10+). These findings suggest that M2 MΦ/MG may potentially impact the progression of CCMs throgh the aforementioned ligand-receptor pathways to interact with tip cells (Mki67+) and drive cuproptosis in tip cells (Mki67+), in which process Btdb10 may play a role in cuproptosis promotion.

## Discussion

This investigation examined the subtypes across CCMs associated with cuproptosis by deploying bioinformatics methods and experimental validation on comprehensive datasets. We discerned two primary subtypes of CCMs intimately associated with the expression of CRGs and screened for hub genes from two subtypes of CCMs. Hub genes were then validated with an external dataset and CCMs samples from our cohort. Additionally, we delved into the potential interactions between hub genes related to cuproptosis and the immune infiltration by scRNA-seq analysis. This comprehensive genomic and bioinformatic investigation provides an invaluable source for understanding mechanisms underpinning the pathogenesis and progression of CCMs, further carving paths for the development of precision therapies for CCMs.

Previous studies have highlighted the presence of immune cells, such as B lymphocytes, plasma cells, T cells, and macrophages, within CCM lesions, indicating an active immune response [43, 44]. In this study, we also found the upregulated DEGs in CCMs samples were primary enriched in immune regulatory pathways, and the activated immune cell population in both CCMs and endothelial samples were significantly increased than those in the normal control group. Inflammatory perturbations in the immune microenvironment have been found to contribute to CCMs pathologic progression, affecting disease outcomes [45]. Additionally, CCMs show an increased expression of inflammation-related genes, proinflammatory cytokines, and chemokines, promoting the recruitment of inflammatory and immune cells [46]. These findings underscore the intricate interplay between immune infiltration and CCM development, suggesting a potential role for immune modulation in CCMs progression.

Cuproptosis, a novel cell death mechanism, has been linked to immune infiltration in various diseases [47–49]. Studies have shown that CRGs play a role in regulating immune cell infiltration in diseases like tuberculosis, breast invasive carcinoma and atherosclerosis. The expression of CRGs has been associated with changes in immune cell profiles, impacting disease progression and patient outcomes. In atherosclerosis, cuproptosis regulators were found to be associated with immune cell infiltration status, highlighting the interplay between cuproptosis, immune responses, and disease development [49]. In this investigation, we partitioned CCMs into two distinct subtypes using consensus clustering analysis based on the expression of CRG. Additionally, these two diverse CCMs subtypes showcase disparities concerning immune infiltration. Cluster 1, brimming with an array of cuproptosis-associated features, is observed to possess a lower extent of immune penetration. Concurrently, we unveiled that the mainstay genes of this cluster exhibit a compelling positive relationship with the anti-inflammatory immune cell type M2 MΦ, a correlation that could stem from its distinct role in exerting negative immune regulatory functions [50]. Hub genes (BTBD10, PFDN4, and CEMIP) representing distinct CCMs subtypes were selected via LASSO regression and random forest, followed by external validation and functional exploration in CCMs. ECs have been proved to play a vital role in vascular function and immune cell infiltration [51]. Activated ECs express adhesion molecules that facilitate interactions with immune cells, leading to immune cell infiltration into the vascular wall [51]. Employing the tool of immunofluorescence co-localization, we discerned that these three distinct genes share a co-localization with ECs in CCM samples. This insinuates the likely possibility that the influx of immune cells may orchestrate the cuproptosis within endothelial cells, guided by hub genes, subsequently playing a possibly significant role within the unfolding narrative of CCMs' progression.

Specifically, our study suggests that BTBD10, one of the hub genes from Cluster 1, may serve as a key regulatory gene of cuproptosis in the development of CCMs. BTBD10 was known to activate AKT by inhibiting PP2A-mediated dephosphorylation and inactivation of AKT [52]. Studies have reported that BTBD10 is a prognostic biomarker in cancer and associated with immune infiltration such as glioma [53], and hepatocellular carcinoma [54, 55]. Furthermore, a decrease in BTBD10 expression has been linked to motor neuron death in cases of amyotrophic lateral sclerosis cases due to the downregulation of the AKT-mediated prosurvival signal [56]. In addition, BTBD10 regulates the progression and apoptosis of pancreatic beta cells through the activation of the AKT signaling pathway [57]. In our previous analysis of signaling pathway, our findings showed that high expression of BTBD10 significantly enhances mTORC1 signaling as well as PI3K-AKT-mTOR signaling. The mTOR signaling pathway if responsible for regulating cell growth and is reported to play a critical role in the formation of CCMs [58]. A study has demonstrated that overexpression of BTBD10 in INS-1 cells could activate of the AKT/mTOR signaling pathway via stimulating phospho-mTOR and AKT, enhancing overall cellular protein translation and promoting the proliferation of INS-1 cells [59]. Furthermore, the correlation analysis revealed a significant correlation between BTBD10 and DLL4, an endothelial Notch ligand which is upregulated by VEGF and regulates tip cells formation during angiogenesis [60, 61]. Tip cells in angiogenesis perform critical roles in sprouting and vessel formation by leading the angiogenic sprouts [62]. In this research, the analysis of scRNA-seq data from the CCMs mice model indicated that Btbd10 is highly expressed in the proliferating tip cells, specifically Mki67+ tip cells. Concurrently, Fdx1, a major gene promoting cuproptosis, are co-expressed with Btbd10 in the Mki67+ tip cells, suggesting that Btbd10 might influence the development of CCMs by participating in the cuproptosis process of Mki67+ tip cells.

M2 MΦ are recognized for their anti-inflammatory properties and have been demonstrated to enhance angiogenesis by promoting tip cell migration and assisting tip cell fusion [63]. On the other hand, MG, the resident MΦ of the brain, have been observed to interact with endothelial tip cells to support vascular anastomosis during brain vascularization [64]. In this study, we identified positive correlations between BDBT10 and M2 MΦ, and negative correlations with M1 MΦ within the Bulk RNA-seq validation dataset from CCMs samples. Intriguingly, our analysis of scRNA-seq data from CCMs mice indicated that the interactions between Mki67+ tip cells with high Btbd10 expression and M2 MΦ/MG are noticeably stronger than those with low Btbd10 expression. These infers a close correlation between the expression of BTBD10 and the interaction with M2 MΦ/MG, suggesting that M2 MΦ/MG may influence the angiogenesis of CCMs potentially by regulating cuproptosis in tip cells through the expression of BTBD10.

Our observation revealed that PFDN4, akin to BTBD10, is sourced from Cluster 1. Moreover, striking similarities can be seen in their expression patterns and relationships with immune cells which pertain closely to those of BTBD10. PFDN4 is known as a subunit of the heterohexameric chaperone protein belonging to the prefoldin family [65, 66]. Its role is to capture unfolded actin and tubulin, delivering them to the cytosolic chaperone [67]. Furthermore, it functions as a transcription factor or cofactor in regulating the cell-cycle[68]. A study has suggested that PFDN4 expression serves to predict the prognosis of colorectal cancer [69]. Furthermore, empirical evidence has supported that PFDN4 may be associated with the progression of cancer progressions [70–72]. Our research has revealed that PFDN4 is enriched in cellular metabolism pathways and the KRAS signaling pathway. Additionally, we have discovered a considerable negative correlation between PFDN4 and ENG, which regulates migration of vascular ECs [73]. In the validation dataset of the human bulk RNA-seq, PFDN4 distinctly parallels M2 MΦ while standing in inverse proportion to M1 MΦ in CCMs. Remarkably, within the scRNA-seq data obtained from mouse CCM specimens, endothelial expressing Pfdn4 at high levels exhibit a stronger interaction intensity with M2 MΦ/MG compared to their interaction with M1 MΦ/MG. This suggests the likelihood of these high-Pfdn4-expressing endothelial impacting through their interaction with M2 MΦ/MG. However, the absence of a defined strong association between Pfdn4 and particular CRGs in the scRNA-seq data set our intense exploration aside for this research, earmarking the elucidation of its role within CCMs for further substantiation in the future.

CEMIP, also known as KIAA1199, was initially identified as a protein in the inner ear, and mutations in its genetic makeup were associated with nonsyndromic hearing loss [74]. Its main role relates to hyaluronan depolymerization [75]. Studies have demonstrated that CEMIP is highly upregulated in various types of cancers and could be a valuable diagnostic and prognostic tool in assessing tumor progression [76, 77]. It has been demonstrated that CEMIP regulates cell proliferation, differentiation, migration, and invasion, promoting tumor growth through activation of pathways including Notch signaling pathway [78], the Wnt signaling pathway [79], and integrin-mediated AKT and ERK-MAPK intracellular signaling [80]. Furthermore, CEMIP acts as an adaptor for the interaction between MHC-I and clathrin, promoting MHC-I internalization via clathrin-dependent endocytosis [81]. In periarticular tissue, inflammatory cytokines induced the expression of CEMIP, which suggests that it may play a significant role in the initiation or development of osteoarthritis [82]. Our findings are consistent with previous research as we have demonstrated that CEMIP expression is predominantly enriched in pathways related to inflammation including IL6/JAK/STAT3 signaling. Simultaneously, a heightened expression of CEMIP is observed in Cluster 2, a subtypes conspicuous for its pronounced level of immune infiltration as revealed in the bulk RNA-seq dataset. Inextricably twined, CEMIP shows a proportional relationship with the pro-inflammatory M1 MΦ. However, owing to the comparatively low expression of CEMIP in the scRNA-seq samples integral to this investigation, the intricate interactions between it and M1 MΦ within CCMs cannot be adequately elucidated. Therefore, it is of paramount importance that further probing investigations are conducted to fully unravel the meticulous mechanisms that govern this gene interactions in CCMs in the future.

There are several limitations in this study. Due to the limited number of available and accessible public datasets for CCMs, coupled with the limited sample size within each dataset, these datasets might not be representative of the entire population of CCMs patients. Lacking a large sample dataset for validation, we resorted to the use of in vitro endothelial bulk RNA-seq dataset from human CCMs for verification. This could result in certain discrepancies with the actual in vivo environment surrounding the lesions. Despite this, the overall results remain consistent with the initial set of cohort data. Moreover, Due to the limited sample size and lack of prognosis-related information, it is not feasible in this study to further ascertain the impact of hub genes on the prognosis. Moving forward, conducting studies with larger sample sizes and more diverse populations would serve to corroborate the findings of this study and enhance the generalizability of the results. Furthermore, even though we discovered that the interactions between MΦ/MG and the ECs in CCMs were intensified, further experiments are still required in the future to validate these phenomena as well as to delve deeper into the precise functions of these three genes in regulating cuproptosis associated to CCMs' angiogenesis.

## Conclusions

In conclusion, we identified two distinct subtypes of CCMs, defined on the basis of CRGs. Additionally, we elucidated three hub genes from different subtypes in ECs associated with cuproptosis that may have substantial implications in the progression of CCMs via the regulation of immune infiltration, thereby presenting potential targets for the treatment of the disease.

### Supplementary Information

Supplementary Material 1

## Data Availability

No datasets were generated or analysed during the current study.

## Abbreviations

CCMs: Cerebral cavernous malformations
RNA-seq: Bulk RNA sequencing
CRGs: Cuproptosis-related genes
ECs: Endothelial cells
FDX1: Ferredoxin 1
LIAS: Lipoic acid synthase
LIPT1: Lipoyltransferase 1
DLD: Dihydrolipoamide dehydrogenase
DLAT: Dihydrolipoamide S-acetyltransferase
PDHA1: Pyruvate dehydrogenase E1 component subunit alpha 1
PDHB: Pyruvate dehydrogenase beta subunit
MTF1: Metal regulatory transcription factor 1
GLS: Glutaminase
CDKN2A: Cyclin-dependent kinase inhibitor 2A
GEO: Gene Expression Omnibus
scRNA-seq: Single-cell RNA-seq
DEGs: Differentially expressed genes
LASSO: Least absolute shrinkage and selection operator
TCA: Tricarboxylic acid cycle
TISIDB: Database of Tumor Immune System Interaction
MHC: Major Histocompatibility Complex
ssGSEA: Single-sample gene set enrichment analysis
CIBERSORT: A computational method for estimating the abundance of different cell types from a bulk tumor transcriptome
GSVA: Gene set variation analysis
GSEA: Gene set enrichment analysis
BCA: Bicinchoninic acid assay
SDS-PAGE: Sodium dodecyl sulfate polyacrylamide gel electrophoresis
ECL: Enhanced chemiluminescence
MsigDB: Molecular Signatures Database
TGFB1: Transforming Growth Factor Beta 1
TNFSF13B: Tumor Necrosis Factor Superfamily Member 13b
HLA-G: Major Histocompatibility Complex, Class I, G
TAP2: Transporter 2, ATP Binding Cassette Subfamily B Member
cAMP: Cyclic adenosine monophosphate
NFκB: Nuclear factor kappa-light-chain-enhancer of activated B cells
GO: Gene Ontology
AKT: Protein kinase B
mTOR: Mechanistic target of rapamycin
PI3K: Phosphoinositide 3-kinase
DLL4: Delta-Like Canonical Notch Ligand 4
ARID1B: AT-Rich Interaction Domain 1B
PIK3R2: Phosphoinositide-3-Kinase Regulatory Subunit 2
NR2F2: Nuclear Receptor Subfamily 2 Group F Member 2
ZIC1: Zic Family Member 1
ENG: Endoglin
aDCs: Activated dendritic cells
DCs: Dendritic cells
Tfh: T follicular helper cell
Treg: Regulatory T cell
TIL: Tumor-Infiltrating Lymphocyte
pDCs: Plasmacytoid dendritic cells
Type I IFN: Type I Interferon
Type II IFN: Type II Interferon
CCR: C-C chemokine receptor
APC: Antigen Presenting Cell
CSF1R: Colony-stimulating factor 1 receptor
IL10: Interleukin 10
PDCD1LG2: Programmed cell death 1 ligand 2
CXCL9: Chemokine (C-X-C motif) ligand 9
CCL22: Chemokine (C-C motif) ligand 22
CXCL12: Chemokine (C-X-C motif) ligand 12
HLA-DOB: Major Histocompatibility Complex, Class II, DO beta
Esam: Endothelial cell-selective adhesion molecule
Col4a1: Collagen type IV alpha 1 chain
Itga4: Integrin alpha 4
Itgb1: Integrin beta 1
Ptn: Pleiotrophin
Ncl: Nucleolin
Fn1: Fibronectin
Btbd10: Broad-complex, tram-track and bric-a-brac domain 10
Mtf1: Metal regulatory transcription factor 1
Gls: Glutaminase
Lipt1: Lipoyltransferase 1
Cdkn2a: Cyclin-dependent kinase inhibitor 2A
Mki67: A protein expressed in proliferating cells
Cemip: Cell migration-inducing protein
PFDN4: Prefoldin 4
