# Supplementary material for: Single-cell and bulk RNA-seq unveils the immune infiltration landscape associated with cuproptosis in cerebral cavernous malformations
Source: Biomark Res. 2024 Jun 5;12:57. doi: 10.1186/s40364-024-00603-y (PMC11151651; doi:10.1186/s40364-024-00603-y)
Supplement: Supplementary file 1 — Supplementary Material 1 [file 40364_2024_603_MOESM1_ESM.pdf]

Supplementary Information

Single-cell and bulk RNA-seq unveils the immune infiltration landscape associated with cuproptosis in cerebral cavernous malformations

Chengwei Chen,[#1,2,3,4,5,6,7] Yuting Bao, [#1,2,3,4,5,6] Sihan Ju,[#1,2,3,4,5,6] Conglin Jiang, [1,2,3,4,5,6] Xiang Zou, [1,2,3,4,5,6] Xin Zhang[1,2,3,4,5.6] and Liang Chen [* 1,2,3,4,5,6]

Author affiliations:

1. Neurosurgical department of Huashan hospital and MOE Frontiers Center for 1 Brain Science, Fudan University, Shanghai 200040, China

2. Tianqiao and Chrissy Chen Institute Clinical Translational Research Center, 2 Shanghai 200040, China

3. National Center for Neurological Disorders, Shanghai 200040, China

4. Shanghai Key Laboratory of Brain Function Restoration and Neural 4 Regeneration, Shanghai 200040, China

5. Neurosurgical Institute of Fudan University, Shanghai 200040, China

6. Shanghai Clinical Medical Center of Neurosurgery, Shanghai 200040, China

7. Neurosurgery Center, Department of Cerebrovascular Surgery, The National 7 Key Clinical Specialty, The Engineering Technology Research Center of Education Ministry of China on Diagnosis and Treatment of Cerebrovascular Disease ,Guangdong Provincial Key Laboratory on Brain Function Repair and Regeneration , The Neurosurgery Institute of Guangdong Province , Zhujiang Hospital , Southern Medical University, Guangzhou 510282, China

#Chengwei Chen,Yuting Bao and Sihan Ju contributed equally to this work.

*Correspondence to

Professor Liang Chen, Huashan Hospital, Shanghai Medical College, Fudan University, Shanghai 200040, China; hschenliang@fudan.edu.cn

Fig. S1

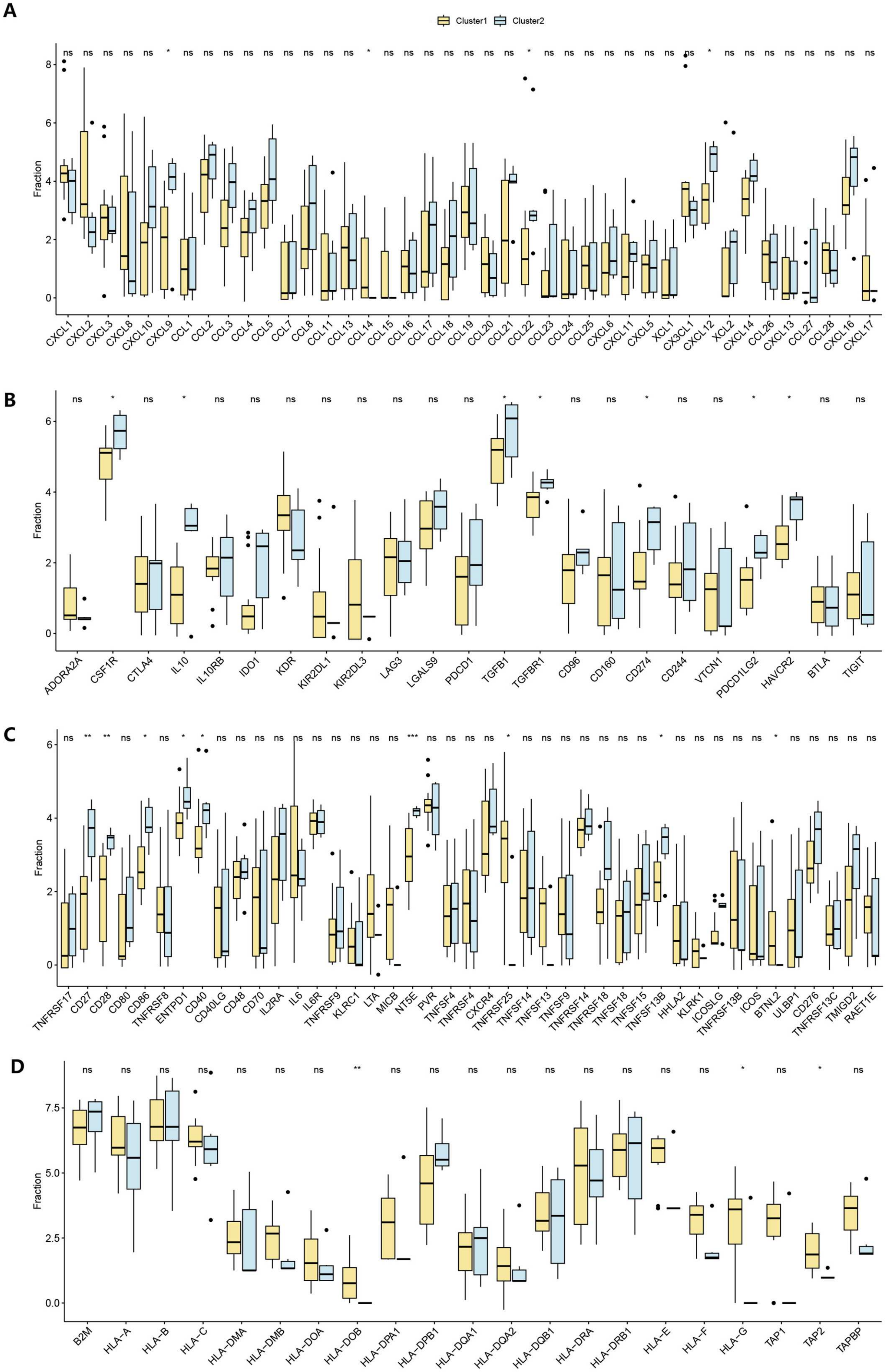
A
Cluster1
Cluster2
Fraction
CXCL1 CXCL2 CXCL3 CXCL8 CXCL10 CXCL9 CCL1 CCL2 CCL3 CCL4 CCL5 CCL7 CCL8 CCL11 CCL13 CCL14 CCL15 CCL16 CCL17 CCL18 CCL19 CCL20 CCL21 CCL22 CCL23 CCL24 CCL25 CXCL6 CXCL11 CXCL5 XCL1 CX3CL1 CXCL12 XCL2 CXCL14 CCL26 CXCL13 CCL27 CCL28 CXCL16 CXCL17
B
Fraction
ADORA2A CSF1R CTLA4 IL10 IL10RB IDO1 KDR KIR2DL1 KIR2DL3 LAG3 LGALS9 PDCD1 TGFB1 TGFBR1 CD96 CD160 CD274 CD244 VTCN1 PDCD1LG2 HAVCR2 BTLA TIGIT
C
Fraction
TNFRSF17 CD27 CD28 CD80 CD86 TNFRSF8 ENTPD1 CD40 CD40LG CD48 CD70 IL2RA IL6 IL6R TNFRSF9 KLRC1 LTA MICB NT5E PVR TNFSF4 TNFRSF4 CXCR4 TNFRSF25 TNFSF14 TNFSF13 TNFSF9 TNFRSF14 TNFRSF18 TNFSF18 TNFSF15 TNFSF13B HHLA2 KLRK1 ICOSLG TNFRSF13B ICOS BTNL2 ULBP1 CD276 TNFRSF13C TMIGD2 RAET1E
D
Fraction
B2M HLA−A HLA−B HLA−C HLA−DMA HLA−DMB HLA−DOA HLA−DOB HLA−DPA1 HLA−DPB1 HLA−DQA1 HLA−DQA2 HLA−DQB1 HLA−DRA HLA−DRB1 HLA−E HLA−F HLA−G TAP1 TAP2 TAPBP

Fig. S2

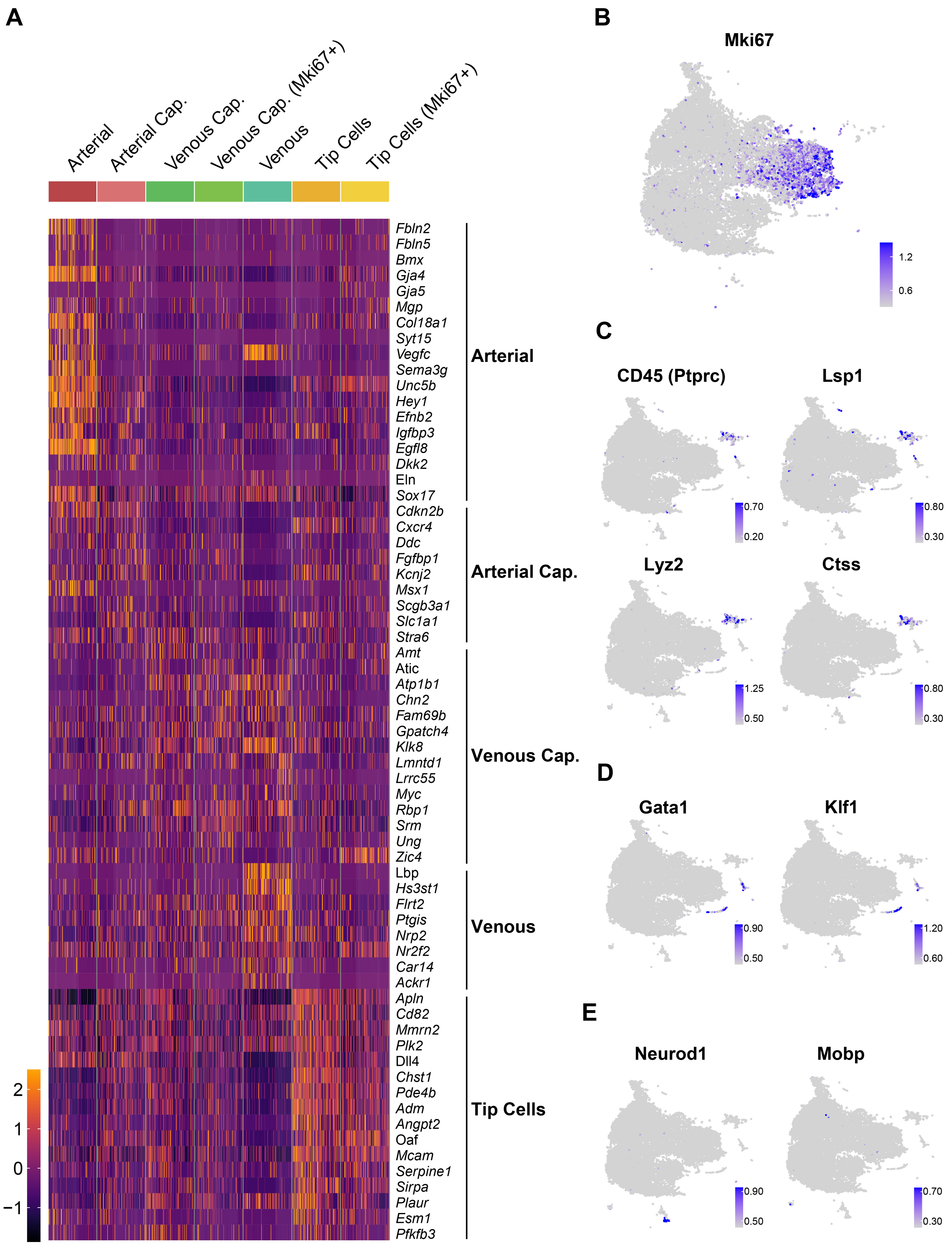

A
Arterial
Arterial Cap.
Venous Cap.
Venous Cap. (Mki67+)
Venous
Tip Cells
Tip Cells (Mki67+)
Fbln2
Fbln5
Bmx
Gja4
Gja5
Mgp
Col18a1
Syt15
Vegfc
Sema3g
Unc5b
Hey1
Efnb2
Igfbp3
Egfl8
Dkk2
Eln
Sox17
Cdkn2b
Cxcr4
Ddc
Fgfbp1
Kcnj2
Msx1
Scgb3a1
Slc1a1
Stra6
Amt
Atic
Atp1b1
Chn2
Fam69b
Gpatch4
Klk8
Lmntd1
Lrrc55
Myc
Rbp1
Srm
Ung
Zic4
Lbp
Hs3st1
Flrt2
Ptgis
Nrp2
Nr2f2
Car14
Ackr1
Apln
Cd82
Mmrn2
Plk2
Dll4
Chst1
Pde4b
Adm
Angpt2
Oaf
Mcam
Serpine1
Sirpa
Plaur
Esm1
Pfkfb3
Arterial
Arterial Cap.
Venous Cap.
Venous
Tip Cells
2
1
0
−1
B
Mki67
1.2
0.6
C
CD45 (Ptprc)
Lsp1
0.70
0.20
0.80
0.30
Lyz2
Ctss
1.25
0.50
0.80
0.30
D
Gata1
Klf1
0.90
0.50
1.20
0.60
E
Neurod1
Mobp
0.90
0.50
0.70
0.30

Fig. S3

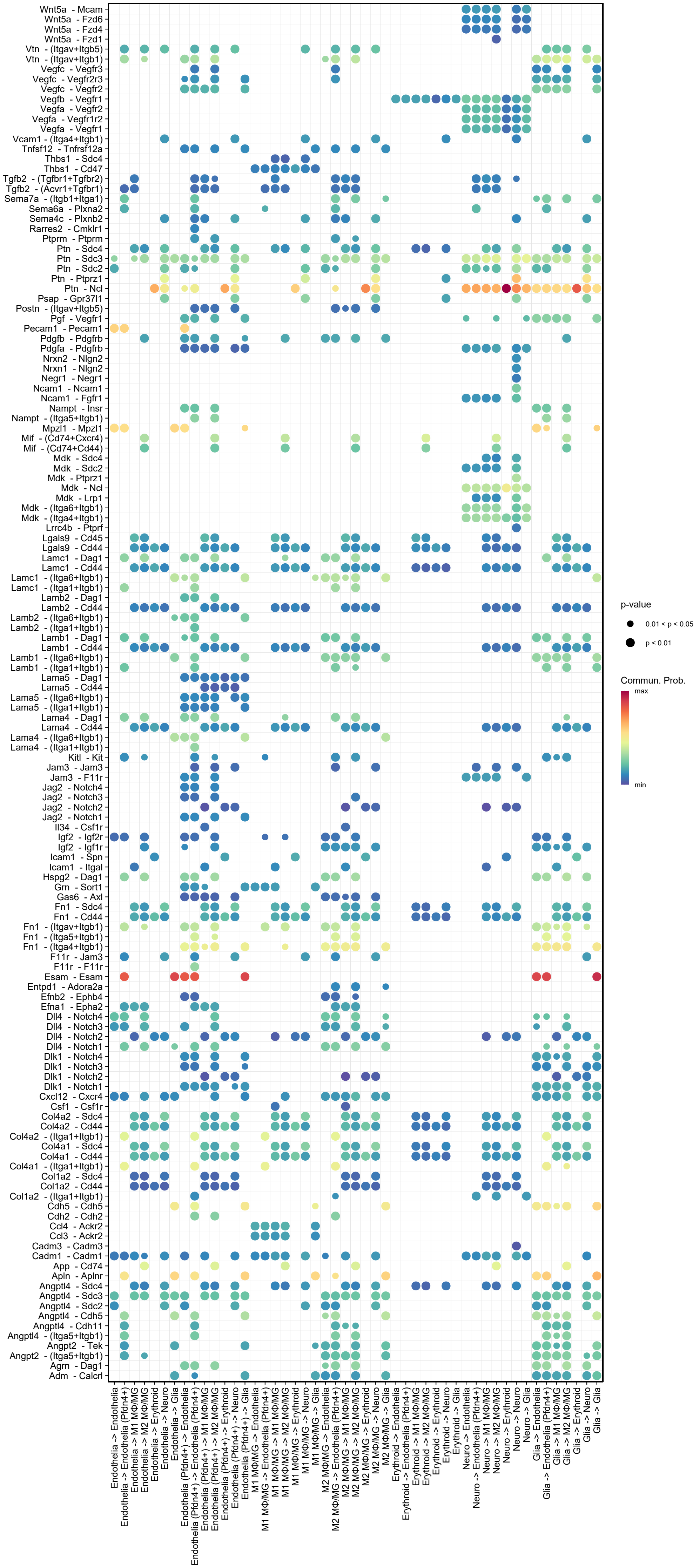
Wnt5a - Mcam
Wnt5a - Fzd6
Wnt5a - Fzd4
Wnt5a - Fzd1
Vtn - (Itgav+Itgb5)
Vtn - (Itgav+Itgb1)
Vegfc - Vegfr3
Vegfc - Vegfr2r3
Vegfc - Vegfr2
Vegfb - Vegfr1
Vegfa - Vegfr2
Vegfa - Vegfr1r2
Vegfa - Vegfr1
Vcam1 - (Itga4+Itgb1)
Tnfsf12 - Tnfrsf12a
Thbs1 - Sdc4
Thbs1 - Cd47
Tgfb2 - (Tgfbr1+Tgfbr2)
Tgfb2 - (Acvr1+Tgfbr1)
Sema7a - (Itgb1+Itga1)
Sema6a - Plxna2
Sema4c - Plxnb2
Rarres2 - Cmklr1
Ptprm - Ptprm
Ptn - Sdc4
Ptn - Sdc3
Ptn - Sdc2
Ptn - Ptprz1
Ptn - Ncl
Psap - Gpr37l1
Postn - (Itgav+Itgb5)
Pgf - Vegfr1
Pecam1 - Pecam1
Pdgfb - Pdgfrb
Pdgfa - Pdgfrb
Nrxn2 - Nlgn2
Nrxn1 - Nlgn2
Negr1 - Negr1
Ncam1 - Ncam1
Ncam1 - Fgfr1
Nampt - Insr
Nampt - (Itga5+Itgb1)
Mpzl1 - Mpzl1
Mif - (Cd74+Cxcr4)
Mif - (Cd74+Cd44)
Mdk - Sdc4
Mdk - Sdc2
Mdk - Ptprz1
Mdk - Ncl
Mdk - Lrp1
Mdk - (Itga6+Itgb1)
Mdk - (Itga4+Itgb1)
Lrrc4b - Ptprf
Lgals9 - Cd45
Lgals9 - Cd44
Lamc1 - Dag1
Lamc1 - Cd44
Lamc1 - (Itga6+Itgb1)
Lamc1 - (Itga1+Itgb1)
Lamb2 - Dag1
Lamb2 - Cd44
Lamb2 - (Itga6+Itgb1)
Lamb2 - (Itga1+Itgb1)
Lamb1 - Dag1
Lamb1 - Cd44
Lamb1 - (Itga6+Itgb1)
Lamb1 - (Itga1+Itgb1)
Lama5 - Dag1
Lama5 - Cd44
Lama5 - (Itga6+Itgb1)
Lama5 - (Itga1+Itgb1)
Lama4 - Dag1
Lama4 - Cd44
Lama4 - (Itga6+Itgb1)
Lama4 - (Itga1+Itgb1)
Kitl - Kit
Jam3 - Jam3
Jam3 - F11r
Jag2 - Notch4
Jag2 - Notch3
Jag2 - Notch2
Jag2 - Notch1
Il34 - Csf1r
Igf2 - Igf2r
Igf2 - Igf1r
Icam1 - Spn
Icam1 - Itgal
Hspg2 - Dag1
Grn - Sort1
Gas6 - Axl
Fn1 - Sdc4
Fn1 - Cd44
Fn1 - (Itgav+Itgb1)
Fn1 - (Itga5+Itgb1)
Fn1 - (Itga4+Itgb1)
F11r - Jam3
F11r - F11r
Esam - Esam
Entpd1 - Adora2a
Efnb2 - Ephb4
Efna1 - Epha2
Dll4 - Notch4
Dll4 - Notch3
Dll4 - Notch2
Dll4 - Notch1
Dlk1 - Notch4
Dlk1 - Notch3
Dlk1 - Notch2
Dlk1 - Notch1
Cxcl12 - Cxcr4
Csf1 - Csf1r
Col4a2 - Sdc4
Col4a2 - Cd44
Col4a2 - (Itga1+Itgb1)
Col4a1 - Sdc4
Col4a1 - Cd44
Col4a1 - (Itga1+Itgb1)
Col1a2 - Sdc4
Col1a2 - Cd44
Col1a2 - (Itga1+Itgb1)
Cdh5 - Cdh5
Cdh2 - Cdh2
Ccl4 - Ackr2
Ccl3 - Ackr2
Cadm3 - Cadm3
Cadm1 - Cadm1
App - Cd74
Apln - Aplnr
Angptl4 - Sdc4
Angptl4 - Sdc3
Angptl4 - Sdc2
Angptl4 - Cdh5
Angptl4 - Cdh11
Angptl4 - (Itga5+Itgb1)
Angpt2 - Tek
Angpt2 - (Itga5+Itgb1)
Agrn - Dag1
Adm - Calcrl
Endothelia -> Endothelia
Endothelia -> Endothelia (Pfdn4+)
Endothelia -> M1 MΦ/MG
Endothelia -> M2 MΦ/MG
Endothelia -> Erythroid
Endothelia -> Neuro
Endothelia -> Glia
Endothelia (Pfdn4+) -> Endothelia
Endothelia (Pfdn4+) -> Endothelia (Pfdn4+)
Endothelia (Pfdn4+) -> M1 MΦ/MG
Endothelia (Pfdn4+) -> M2 MΦ/MG
Endothelia (Pfdn4+) -> Erythroid
Endothelia (Pfdn4+) -> Neuro
Endothelia (Pfdn4+) -> Glia
M1 MΦ/MG -> Endothelia
M1 MΦ/MG -> Endothelia (Pfdn4+)
M1 MΦ/MG -> M1 MΦ/MG
M1 MΦ/MG -> M2 MΦ/MG
M1 MΦ/MG -> Erythroid
M1 MΦ/MG -> Neuro
M1 MΦ/MG -> Glia
M2 MΦ/MG -> Endothelia
M2 MΦ/MG -> Endothelia (Pfdn4+)
M2 MΦ/MG -> M1 MΦ/MG
M2 MΦ/MG -> M2 MΦ/MG
M2 MΦ/MG -> Erythroid
M2 MΦ/MG -> Neuro
M2 MΦ/MG -> Glia
Erythroid -> Endothelia
Erythroid -> Endothelia (Pfdn4+)
Erythroid -> M1 MΦ/MG
Erythroid -> M2 MΦ/MG
Erythroid -> Erythroid
Erythroid -> Neuro
Erythroid -> Glia
Neuro -> Endothelia
Neuro -> Endothelia (Pfdn4+)
Neuro -> M1 MΦ/MG
Neuro -> M2 MΦ/MG
Neuro -> Erythroid
Neuro -> Neuro
Neuro -> Glia
Glia -> Endothelia
Glia -> Endothelia (Pfdn4+)
Glia -> M1 MΦ/MG
Glia -> M2 MΦ/MG
Glia -> Erythroid
Glia -> Neuro
Glia -> Glia
p-value
0.01 < p < 0.05
p < 0.01
Commun. Prob.
max
min

Fig. S4

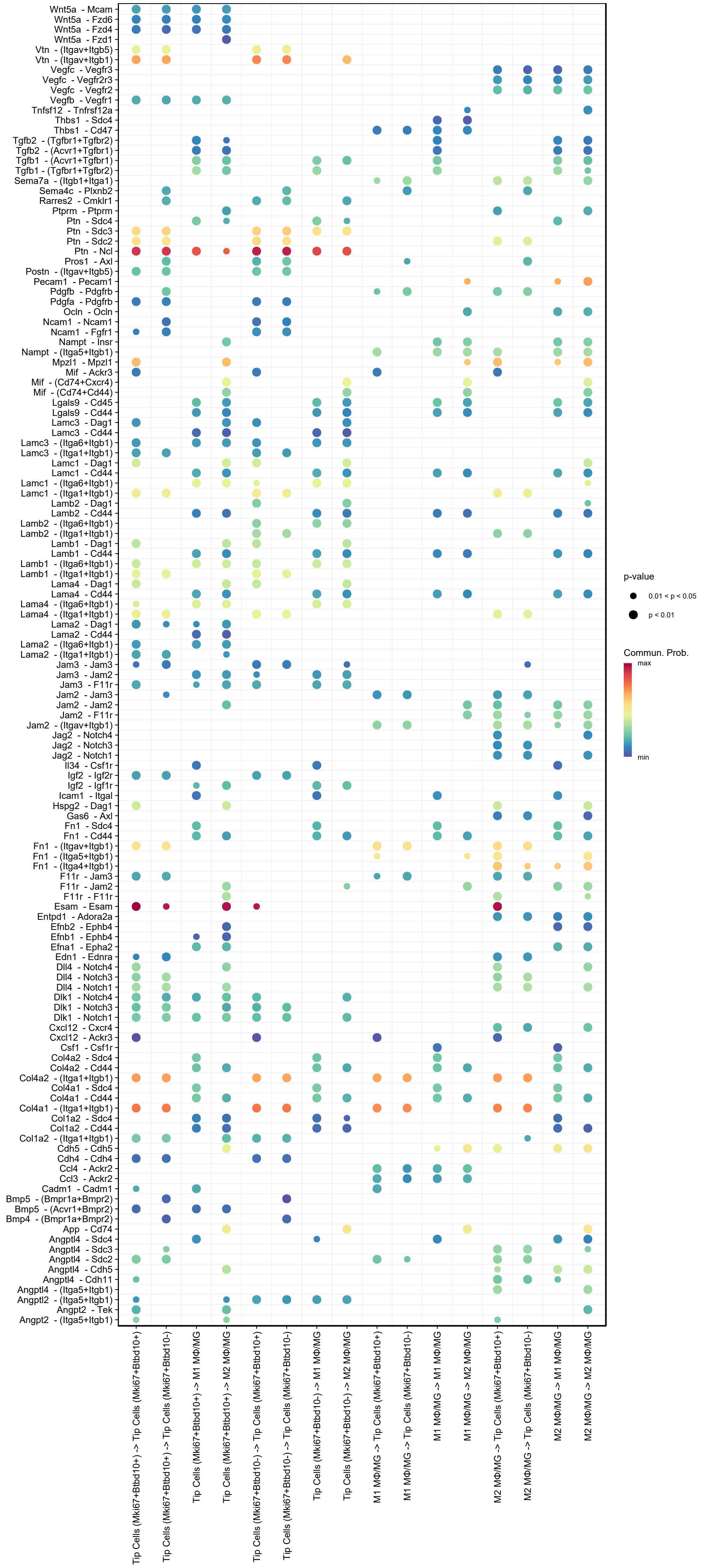
Wnt5a - Mcam
Wnt5a - Fzd6
Wnt5a - Fzd4
Wnt5a - Fzd1
Vtn - (Itgav+Itgb5)
Vtn - (Itgav+Itgb1)
Vegfc - Vegfr3
Vegfc - Vegfr2r3
Vegfc - Vegfr2
Vegfb - Vegfr1
Tnfsf12 - Tnfrsf12a
Thbs1 - Sdc4
Thbs1 - Cd47
Tgfb2 - (Tgfbr1+Tgfbr2)
Tgfb2 - (Acvr1+Tgfbr1)
Tgfb1 - (Acvr1+Tgfbr1)
Tgfb1 - (Tgfbr1+Tgfbr2)
Sema7a - (Itgb1+Itga1)
Sema4c - Plxnb2
Rarres2 - Cmklr1
Ptprm - Ptprm
Ptn - Sdc4
Ptn - Sdc3
Ptn - Sdc2
Ptn - Ncl
Pros1 - Axl
Postn - (Itgav+Itgb5)
Pecam1 - Pecam1
Pdgfb - Pdgfrb
Pdgfa - Pdgfrb
Ocln - Ocln
Ncam1 - Ncam1
Ncam1 - Fgfr1
Nampt - Insr
Nampt - (Itga5+Itgb1)
Mpzl1 - Mpzl1
Mif - Ackr3
Mif - (Cd74+Cxcr4)
Mif - (Cd74+Cd44)
Lgals9 - Cd45
Lgals9 - Cd44
Lamc3 - Dag1
Lamc3 - Cd44
Lamc3 - (Itga6+Itgb1)
Lamc3 - (Itga1+Itgb1)
Lamc1 - Dag1
Lamc1 - Cd44
Lamc1 - (Itga6+Itgb1)
Lamc1 - (Itga1+Itgb1)
Lamb2 - Dag1
Lamb2 - Cd44
Lamb2 - (Itga6+Itgb1)
Lamb2 - (Itga1+Itgb1)
Lamb1 - Dag1
Lamb1 - Cd44
Lamb1 - (Itga6+Itgb1)
Lamb1 - (Itga1+Itgb1)
Lama4 - Dag1
Lama4 - Cd44
Lama4 - (Itga6+Itgb1)
Lama4 - (Itga1+Itgb1)
Lama2 - Dag1
Lama2 - Cd44
Lama2 - (Itga6+Itgb1)
Lama2 - (Itga1+Itgb1)
Jam3 - Jam3
Jam3 - Jam2
Jam3 - F11r
Jam2 - Jam3
Jam2 - Jam2
Jam2 - F11r
Jam2 - (Itgav+Itgb1)
Jag2 - Notch4
Jag2 - Notch3
Jag2 - Notch1
Il34 - Csf1r
Igf2 - Igf2r
Igf2 - Igf1r
Icam1 - Itgal
Hspg2 - Dag1
Gas6 - Axl
Fn1 - Sdc4
Fn1 - Cd44
Fn1 - (Itgav+Itgb1)
Fn1 - (Itga5+Itgb1)
Fn1 - (Itga4+Itgb1)
F11r - Jam3
F11r - Jam2
F11r - F11r
Esam - Esam
Entpd1 - Adora2a
Efnb2 - Ephb4
Efnb1 - Ephb4
Efna1 - Epha2
Edn1 - Ednra
Dll4 - Notch4
Dll4 - Notch3
Dll4 - Notch1
Dlk1 - Notch4
Dlk1 - Notch3
Dlk1 - Notch1
Cxcl12 - Cxcr4
Cxcl12 - Ackr3
Csf1 - Csf1r
Col4a2 - Sdc4
Col4a2 - Cd44
Col4a2 - (Itga1+Itgb1)
Col4a1 - Sdc4
Col4a1 - Cd44
Col4a1 - (Itga1+Itgb1)
Col1a2 - Sdc4
Col1a2 - Cd44
Col1a2 - (Itga1+Itgb1)
Cdh5 - Cdh5
Cdh4 - Cdh4
Ccl4 - Ackr2
Ccl3 - Ackr2
Cadm1 - Cadm1
Bmp5 - (Bmpr1a+Bmpr2)
Bmp5 - (Acvr1+Bmpr2)
Bmp4 - (Bmpr1a+Bmpr2)
App - Cd74
Angptl4 - Sdc4
Angptl4 - Sdc3
Angptl4 - Sdc2
Angptl4 - Cdh5
Angptl4 - Cdh11
Angptl4 - (Itga5+Itgb1)
Angptl2 - (Itga5+Itgb1)
Angpt2 - Tek
Angpt2 - (Itga5+Itgb1)
Tip Cells (Mki67+Btbd10+) -> Tip Cells (Mki67+Btbd10+)
Tip Cells (Mki67+Btbd10+) -> Tip Cells (Mki67+Btbd10-)
Tip Cells (Mki67+Btbd10+) -> M1 MΦ/MG
Tip Cells (Mki67+Btbd10+) -> M2 MΦ/MG
Tip Cells (Mki67+Btbd10-) -> Tip Cells (Mki67+Btbd10+)
Tip Cells (Mki67+Btbd10-) -> Tip Cells (Mki67+Btbd10-)
Tip Cells (Mki67+Btbd10-) -> M1 MΦ/MG
Tip Cells (Mki67+Btbd10-) -> M2 MΦ/MG
M1 MΦ/MG -> Tip Cells (Mki67+Btbd10+)
M1 MΦ/MG -> Tip Cells (Mki67+Btbd10-)
M1 MΦ/MG -> M1 MΦ/MG
M1 MΦ/MG -> M2 MΦ/MG
M2 MΦ/MG -> Tip Cells (Mki67+Btbd10+)
M2 MΦ/MG -> Tip Cells (Mki67+Btbd10-)
M2 MΦ/MG -> M1 MΦ/MG
M2 MΦ/MG -> M2 MΦ/MG
p-value
0.01 < p < 0.05
p < 0.01
Commun. Prob.
max
min

Figure legends

Fig. S1 Comparison of immune system interactions in two clusters. Comparisons of immune infiltration of (**A**) immune-related chemokines, (**B**) immunosuppressive factors, (**C**) immunostimulatory factors and (**D**) MHC between the Cluster 1 and Cluster 2. Values are represented as the mean ± SD. *P*-values are denoted by asterisks: **P* < 0.05, ***P* < 0.01, ****P* < 0.001 and ns indicates no significant difference.

Fig. S2 Expression profiling of marker genes across identified cell types. (**A**) A heatmap showcases the expression of canonical markers for 6 endothelial subtypes, with color gradients indicating expression levels; (**B- E**) UMAP plots depict the expression of proliferation marker (Mki67), Mφ/MG markers (Ptprc, Lsp1, Lyz2, Ctss), erythroid markers (Gata1, Klf1), and neuro/glia markers (Neurod1, Mobp) across all identified cell types.

Fig. S3 A dotplot displays the enriched pathways, receptors, and ligands involved in cellchat communication between different cell clusters. *P*-value was indicated by the size of the dot, with smaller *P*-values represented by larger dots. Communicate probability of cellchat was presented using a color bar, where shades closer to red represent a higher probability, and shades closer to blue represent a lower probability. MΦ, macrophage; MG, microglia.

Fig. S4 A dotplot displays the enriched pathways, receptors, and ligands involved in cellchat communication between M2 MΦ/MG and Tip cell (Mki67+Btbd10±). *P*-value was indicated by the size of the dot, with smaller *P*-values represented by larger dots. Communicate probability of cellchat was presented using a color bar, where shades closer to red represent a higher probability, and shades closer to blue represent a lower probability. MΦ, macrophage; MG, microglia.
